# Transcriptional Analysis of Resistance to Low Temperatures in Bermudagrass Crown Tissues

**DOI:** 10.1371/journal.pone.0136433

**Published:** 2015-09-08

**Authors:** Kalpalatha Melmaiee, Michael Anderson, Sathya Elavarthi, Arron Guenzi, Patricia Canaan

**Affiliations:** 1 Department of Plant and Soil Sciences, Oklahoma State University, Stillwater, Oklahoma, United States of America; 2 Department of Biochemistry and Molecular Biology, Oklahoma State University, Stillwater, Oklahoma, United States of America; Università della Calabria, ITALY

## Abstract

Bermudagrass (*Cynodon dactylon* L pers.) is one of the most geographically adapted and utilized of the warm-season grasses. However, bermudagrass adaptation to the Northern USA is limited by freeze damage and winterkill. Our study provides the first large-scale analyses of gene expression in bermudagrass regenerative crown tissues during cold acclimation. We compared gene expression patterns in crown tissues from highly cold tolerant “MSU” and susceptible “Zebra” genotypes exposed to near-freezing temperatures. Suppressive subtractive hybridization was used to isolate putative cold responsive genes Approximately, 3845 transcript sequences enriched for cold acclimation were deposited in the GenBank. A total of 4589 ESTs (3184 unigenes) including 744 ESTs associated with the bermudagrass disease spring dead spot were printed on microarrays and hybridized with cold acclimated complementary Deoxyribonucleic acid (cDNA). A total of 587 differentially expressed unigenes were identified in this study. Of these only 97 (17%) showed significant NCBI matches. The overall expression pattern revealed 40% more down- than up-regulated genes, which was particularly enhanced in MSU compared to Zebra. Among the up-regulated genes 68% were uniquely expressed in MSU (36%) or Zebra (32%). Among the down-regulated genes 40% were unique to MSU, while only 15% to Zebra. Overall expression intensity was significantly higher in MSU than in Zebra (p value ≤ 0.001) and the overall number of genes expressed at 28 days was 2.7 fold greater than at 2 days. These changes in expression patterns reflect the strong genotypic and temporal response to cold temperatures. Additionally, differentially expressed genes from this study can be utilized for developing molecular markers in bermudagrass and other warm season grasses for enhancing cold hardiness.

## Introduction

Bermudagrass, *Cynodon dactylon* L Pers., is one of the most important warm-season perennial turf and forage grasses in use today and is widely adapted across a range of climatic zones extending between 45° north to 45° south latitudes [1,2]. A major limitation to bermudagrass cultivation is susceptibility to freeze damage, particularly in the more northerly and southerly areas of adaptation. Bermudagrass varieties exhibit a wide range of tolerance to cold temperatures with LT_50_ values (the temperature at which 50% of the plants survive) ranging from -4.8° to -11.5°C [3,4]. This range suggests opportunities for further improvement through breeding or genetic engineering approaches.

Bermudagrass’s ability to adapt to more temperate climates depends on its ability to cold acclimate. With the advent of cold temperatures leaves, stems, and roots die back leaving crown and rhizome regenerative tissues as the only living remnant. Winter survival with subsequent spring emergence and regeneration depends on the cold survival of these remnant tissues. In the advanced stages of cold acclimation, crown tissues undergo what might be referred to as a metabolically-inactive quiescent state, possibly related to dormancy.

Many plants adapt to cold temperatures through a metabolically driven acclimation process initiated by a primary cold temperature exposure [5–7]. Cold acclimation typically involves the coordination and expression of hundreds of cold regulated genes [6,8]. Acclimation adjustments at the molecular level were observed in many plant species which include: 1) production of apoplastic antifreeze proteins that retard the growth of lethal ice crystals during freezing conditions [9–11], 2) changes in membrane fluidity by increasing fatty acid saturation [12], 3) reduction in photosynthetic rates leading to reduced production of reactive oxygen [6,13–15], 4) induction of antioxidants to reduce oxidative damage [16], 5) accumulation of cold-regulated (COR) proteins [17,18] and 6) induction of isozymes [19,20] which function at lower temperatures. Many of these mechanisms are well established in a number of acclimating plant species, but little is known in cold acclimating warm season perennials.

Additionally, molecular changes observed in acclimation of bermudagrass crowns to non-freezing temperatures over a period of time include reduction in electrolyte leakage [21], accumulation of COR and PR proteins [22] and the induction of chitinase genes [23]. Recently, more detailed works highlight changes in bermudagrass carbohydrates, proline, total proteins, photochemical efficiency, abscisic acid (ABA) and cytokinin content, dehydrin accumulation, and antioxidant enzyme expression in response to cold temperatures [24–28]. These attributes can be an indication of cold acclimation.

A majority of the research in bermudagrass has been focused on one or few genes, proteins or physiological processes at a time [12,24,26,27,29]. Little is known about gene expression adaptation to cold temperatures on a genomic scale. Genomic approaches have yielded increased insight into molecular adaptation to cold temperature in *Arabidopsis* [30,31] and other species [32,33]. Furthermore, since resistance to spring dead spot (a major bermudagrass disease) is correlated with cold tolerance possibly indicating shared resistance mechanisms [34], we decided to include expression sequence taqs (ESTs) associated with spring dead spot treatments in this investigation. The objective of this research is to identify genes in bermudagrass crown tissues that are differentially expressed and specifically associated with cold tolerance during early and late acclimation responses using microarray technologies.

## Materials and Methods

### Bermudagrass genotypes

The collection of bermudagrass germplasm at Oklahoma State University is among the largest in the world. Many genotypes within the collection have been extensively evaluated for freezing tolerance, with MSU being the most tolerant and Zebra the most susceptible. MSU was collected from the campus of Michigan State University in the winter, and Zebra originated as an F_1_ mutant exhibiting variegated chlorotic leaf striping [35]. Both are tetraploid (2n = 36), produce seed and were maintained under greenhouse and vegetative conditions. Significantly, Zebra will not over-winter when grown in field plots in Stillwater, Oklahoma due to its lack of cold tolerance. MSU and Zebra exhibited LT_50_ values of −10.0°C and −6.0°C, respectively [1].

### Cold acclimation and tissue collection

The procedure for cold acclimating bermudagrass crown tissues was followed as described elsewhere [23]. Mature phytomers from each genotype were transplanted into sixty 14 cm diameter pots, and maintained for a period of two months in the greenhouse. After two months of growth, plants were transferred to two separate growth chambers each containing 30 pots (15 pots for each genotype). Both chambers were set for a 10 h photoperiod. The temperatures of growth chamber containing control plants was set at 28°C day and 24°C night while the chamber containing cold-treated material was set at 8°C day and 2°C night. After 2 and 28 days, samples were collected around noon. All harvesting and processing of plant materials for microarray analysis was performed in a cold room at 4°C. Cold treated plants were gently removed from the pots, roots were cleaned thoroughly with ice-cold water to avoid any warm-up during processing. Plant materials for each genotype were pooled and crown tissues were separated, within 10 minutes, and soaked overnight in RNAlater (Ambion/Life Technologies, USA) at 4°C. Tissues were then blotted dry, weighed, and divided approximately into 2.0 grams samples. These samples were wrapped in an aluminum foil as sample pockets, flash- frozen in liquid nitrogen and stored at –80°C for RNA isolation. Each of these pockets served as a biological replication for downstream analysis. Crown tissues from control plants were harvested and processed as described above except at room temperature and without the ice cold rinse [36].

### Suppression Subtractive Hybridization (SSH) Library creation

Total RNA was extracted from crown tissues with Fenozol reagent (Active Motif, USA), and mRNA was isolated from the total RNA using the mTRAP Total mRNA Isolation kit (Active Motif, USA). This protocol yielded high quality mRNA, largely free of ribosomal RNA, proteins and genomic DNA. Subtracted libraries were constructed by following the Diatchenko’s method [37] and Clontech PCR-Select cDNA Subtraction kit (Clonetech, USA). Both forward (cDNA from control sample was used to subtract from cDNA of cold acclimated sample) and reverse subtractions (cDNA from cold acclimated sample was used to subtract from cDNA of control sample) were performed for each genotype and at each time point (2- and 28- days) to enrich for polymerase chain reaction (PCR) amplicons representative of both up- and down- regulated genes during cold acclimation yielding eight libraries in total (S1 Fig). These subtractions were performed only within genotypes to enrich for the genes altered by treatment. PCR products for each subtraction were inserted and ligated into plasmids using a Qiagen PCR cloning Plus kit and transformed into Qiagen EZ competent cells (Qiagen, USA). These were selected for positive inserts by blue-white screening and archived at -80°C in freezing media (15% glycerol). All clones were screened for inserts by PCR amplification followed by agarose gel electrophoresis, eliminating those with insert sizes less than 200 bp. Inserts were sequenced, vector sequences trimmed using the Pipeonline 3.0 processing and functional sorting software [38]. A total of 3845 high quality sequences were obtained. Eight libraries were created including forward and reverse subtracted libraries for each of the four treatments.

### Microarray printing and hybridization

Plasmid (pDrive Cloning Vector with SSH products) purification and DNA preparation was performed using a Biomek 200 workstation (Beckman Coulter, USA) using an in-house protocol (http://www.noble.org/research/genomics/protocol-biomek/) in order to amplify the inserts. Along with the 3845 clones obtained from the current cold acclimation isolations, 744 EST clones from a subtracted library containing differentially expressed genes in response to the fungal disease spring dead spot infection [24] were included in the overall library adding up to a total of 4589 clones represented by 3184 unigenes. Inserts from purified plasmids were PCR amplified with M13Forward (5’-TGTAAAACGACGGCCAGT-3’) and M13Reverse (5’-TCACAGAGGAAACAGCTATGAC-3’) primers using MangoTaq DNA polymerase (Bioline, USA), ethanol precipitated, concentration and purity and integrity were checked using a NanoDrop spectrophotometer (Thermo Fisher, USA) and by agarose gel electrophoresis. The lengths of these products ranged from 200-500bp. The Arabidopsis Functional Genomics Consortium (AFGC) microarray controls were obtained from Nottingham Arabidopsis Stock Center (http://arabidopsis.org.uk) consisting of 8 transgenic and 10 spiking controls which were included in the microarray printing. Bermudagrass actin, 18S rRNA, chitinase, and wheat actin genes were used as internal controls. Purified DNA from samples and controls were printed on Arrayit Superamine2 slides (Arrayit Corp, USA). A total of 4611 ESTs including controls were printed with three replications using the Omnigrid-100 Microarrayer (Digilab, USA) for a total of 13,833 spots on each slide.

For preparing microarray targets, plants grown, experimental conditions maintained, crown tissues collected, total RNA and mRNA isolated as mentioned earlier but in a separate experiment. Microarray hybridizations were performed with cDNA prepared from 1.5 μg of mRNA each from cold acclimated and control tissues for both genotypes and time points. cDNA labelling was accomplished by reverse transcription of mRNA using dye specific RNA-RT primer mix with Superscript II and nucleotides labeled with either Alexafluor 546 for control (non-acclimated) and Alexafluor 647 for treated (acclimated) mRNA (Invitrogen/ Life Technologies, USA). cDNA hybridization mix was prepared by combining the control and cold acclimated cDNA along with formamide-based hybridization buffer and LNA dT blocker (Genisphere, USA) and hybridizing in 3x SSC solution at 50°C overnight. The slides were washed, rinsed and spun dry. Corresponding 3DNA capture reagents, AF546 and AF647 were hybridized in the darkroom according to the Array 900 kit protocol (Genisphere, USA). The microarray hybridizations were replicated three times using mRNA samples from biological replications for each time point and genotype separately yielding 12 hybridizations. A dye swapping experiment was performed for the Zebra 2 day cDNA in order to investigate dye bias. Self on self was performed for MSU 28 day samples and there was no bias detected.

### Microarray data analysis

The LIMMA (Linear Models for Microarray Data) module [39] was used through the Bioconductor software (www.bioconductor.org) for microarray data analysis. The following statistical parameters were applied during analysis: Robust regression for background correction, Loess normalization for pin-by-pin intensity dependant within-array normalization, Quantile procedure for normalization between arrays, false discovery rate control using P value statistic, empirical Bayes approach using B-statistics (EBayes). ESTs that showed at least 2-fold change in expression with cold treated cDNA when compared to non-treated cDNA with p value < 0.001 were considered differentially expressed in this study. Based on the above criteria, there were 1470 differentially expressed ESTs.

Differentially expressed ESTs were assembled using the CAP3 software (http://genome.cs.mtu.edu/sas.html) resulting in 104 contigs and 482 singlets for a total of 586 differentially expressed unigenes. These unigenes were compared with the Universal Protein Resource (UniProt, www.pir.uniprot.org) and NCBI databases using basic local alignment search tool (BLASTx). Significant similarities were confirmed for genes with an e-value less than 0.01. A four way Venn diagram was constructed using the online tool Venny 2.0 (http://bioinfogp.cnb.csic.es/tools/venny/index.html). Average expression values were calculated in Excel (Microsoft, USA) and statistically analysed using Analysis of Variance (SAS-JMP-Pro v11.0, USA).

Gene Ontology (GO) terms were linked to functional characteristics using the arabidopsis information resource (TAIR) gene ontology search function (http://www.arabidopsis.org/tools/bulk/go/index.jsp) for the differentially expressed genes. GO functions were annotated for the differentially expressed genes obtained from microarray analysis. GO uses a standardized gene vocabulary embedded in a hierarchal relational system to annotate gene function. We use the GO Slim function from the TAIR site to place each gene within this gene ontology framework. The GO Slim function categorizes gene function in three main domains including: biological processes (general pathways), molecular functions (interactions at the molecular level) and cellular components (cellular or organelle relationships). Descriptions of each domain are available at: http://www.arabidopsis.org/help/helppages/go_slim_help.jsp. The overall assessment using the slimmer functions reflect the major areas affected by cold temperatures. The functional characterization data was assembled into cellular, molecular and biological process domains and distinguished as either up, down or mixed regulated responses. GO functional analysis allows for the identification of functional domains impacted by cold acclimation and exposure in bermudagrass crown tissues.

### Real-Time Quantitative Reverse Transcription PCR (qRT-PCR)

A total of 1.5 μg of total RNA was treated with DNAse I and a 2 μLaliquot was used for reverse transcription with SuperScript II Reverse Transcriptase (Invitrogen/Life Technologies, USA). Optimal primer concentrations were determined and PCR reactions performed using 2 μL of 1/25 diluted cDNA, and reverse transcriptase reagents in a final volume of 20 μL. The primer sequences utilized were mentioned in the Table 1. Quantitative RT PCR was performed in an ABI PRSIM 7300 thermal cycler (Applied Biosystems/Life Technologies, USA) using relative quantification settings and SYBR green dye, and the results were analysed using the ABI PRISM 7300 SDS software. This process was repeated for all three biological replications. The auto-ct function was used to calculate threshold cycle (CT) values and the values were averaged from two duplicate runs as technical replications. Normalization of gene expression was performed by subtracting internal control (18S ribosomal RNA) expression values. ΔΔC_T_-values were calculated [40] and values were transformed to log_2_ for microarray data comparison.

**Table 1:** Primer sequences utilized for Real-Time Quantitative Reverse Transcription PCR (qRT-PCR).

S.N	NCBI accession number	Primer sequences
1	BQ826306	Forward: GCTCTGCTTGTGGACTTCTT
		Reverse: AGTAGAGCTTCCAGGTGTATTTC
2	BQ826279	Forward: CAGGCCTCCTTGTGAAATCT
		Reverse: CTGGTCTGCACATTGATCCT
3	BQ825934	Forward: CATTGCCTCTTCCTTCGATTTG
		Reverse: AAGCCTCTGTTGGAGATGTG
4	BQ826356	Forward: GGCAGCATCACGAGAAAGTA
		Reverse: CCGAAGAAGCCCTTGAAGAA
5	18s rRNA	Forward: TGGCGTCAAGGAGAACTAATG
		Reverse: GTTGCCGAGAGTCATGTGAT

## Results and Discussion

With the onset of fall and the coming of cold and freezing temperatures, bermudagrass shoot and root tissue dies back leaving the crown as the major regenerative source for future spring regrowth. The ability of bermudagrass to regenerate in the spring depends on the crown tissues ability to maintain energy reserves and resist prolonged cold temperatures [41]. Warm season bermudagrass crown tissues exposed to cold temperatures were hypothesized to produce significant changes in gene expression in support of the low temperature survival and the cold acclimation process. Here we examine the changes after 2 and 28 days of cold treatment in resistant and susceptible cultivars of bermudagrass. PCR based suppression subtractive libraries were constructed from cold acclimated and non-acclimated crown tissues of MSU and Zebra resulting in 3845 enriched ESTs all of which were deposited in GenBank (Accession numbers: DN989227.1 to DN985375.1). Microarray analysis of these ESTs resulted in the discovery of 586 differentially expressed unigenes (S1 Table), or 19% of the total unigenes (3184) on the array. Of these, only 17% (97 genes) showed significant similarity (e value >0.001) to sequences in the NCBI database. Of the 97 genes, 48 were up, 43 down and 6 from mixed regulated genes (S2 Table). Most likely the lack of known matches is due to: 1) fewer *Cynodon* sp. or *Chloridoidea* subfamily genes available in the NCBI database, 2) fewer genes from crown or other regenerative tissues compared to leaf, stem or root tissues, 3) a lack of knowledge and research efforts concerning genes associated with cold acclimation conditions in warm season perennials.

**Fig 1:**
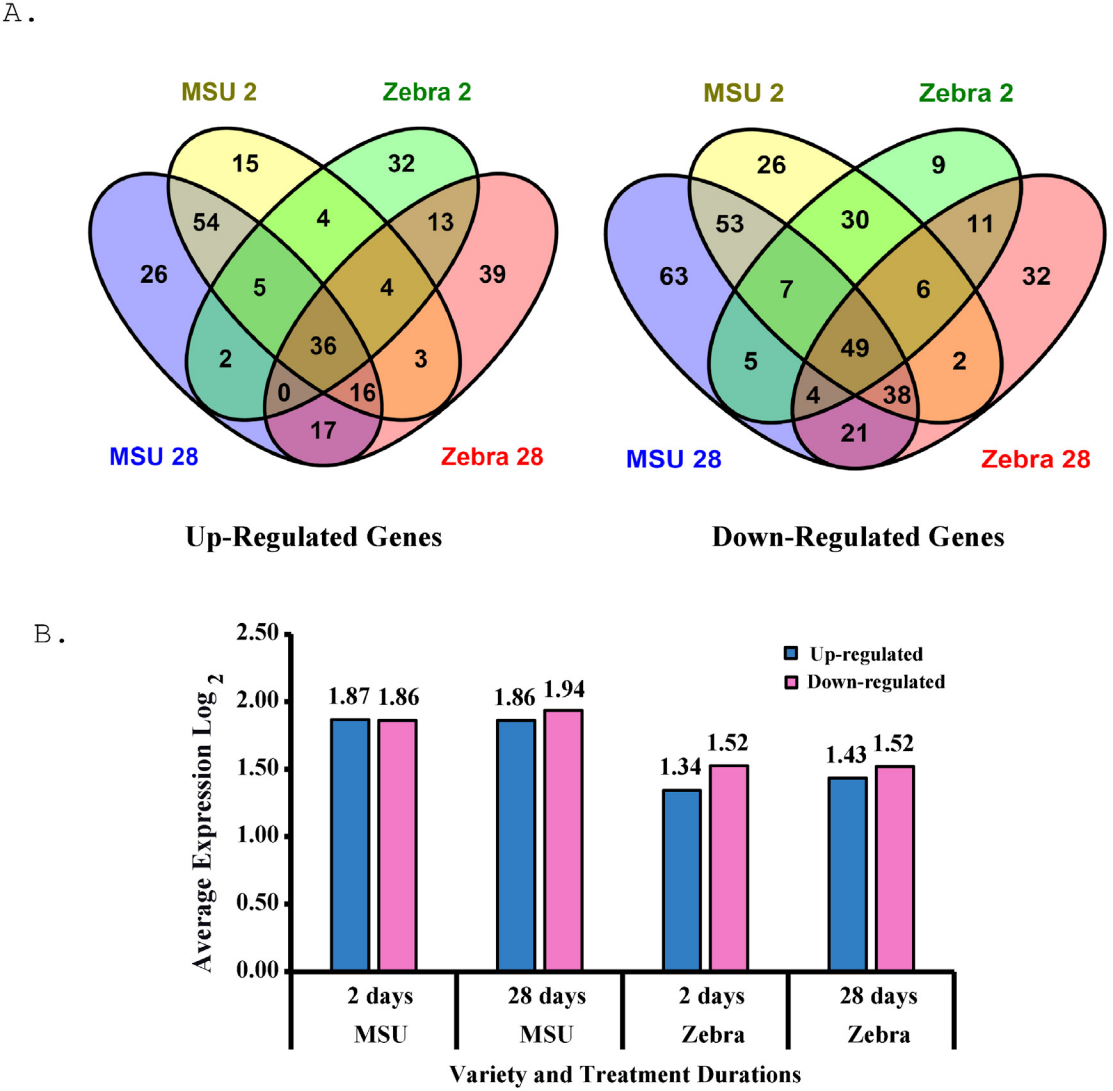
Differential gene expression in resistant and susceptible bermudagrass exposed to cold temperatures for 2 and 28 days. **A** Venn diagram showing overlapping and unique genes in MSU and Zebra, **B** Average up and down expression values for each genotype and cold acclimation treatment.

Examining the overall numbers of genes expressed during cold treatment showed a total of 40% more down than up-regulated genes consistent with gene suppression during cold acclimation in crown tissues. This was more evident in resistant MSU than in susceptible Zebra where the number of down-regulated genes were 53% and 27% greater than up-regulated genes, respectively (data not shown). Further insight is gained by examining the effect of specific treatment combinations on expression as illustrated in the Venn diagrams in Fig 1A. Of all gene expression combinations among treatments, 67% of the up-regulated genes were either unique to MSU (36%) or Zebra (32%) revealing a very large genotype specific involvement in response to cold temperatures. The greatest difference between genotype was shown by the expression of genes common across 2 and 28 days of acclimation where MSU expressed 54 while Zebra only 13 genes, a 4.1 fold difference, indicating that MSU up-regulation genetic response is less influenced by temporal effects than is Zebra. Overall, 36 genes (13% of total) were conserved across genotypes and treatment durations. These conserved genes are likely to be important to the basic cold acclimation process in general. The number of up-regulated genes increased modestly by 38% from 2 to 28 days of acclimation.

Where as 142 (40%) down-regulated genes were unique to MSU, while only 52 (15%) were unique to Zebra indicating a large genotype specific down-regulation response. The down-regulations across 2 and 28 days of acclimation was dramatically in favour of MSU vs Zebra with 53 genes vs 11 genes respectively, a 4.8 fold difference, again indicating that MSU down-regulated response is much less influenced by temporal associations. Furthermore the specific genotypic response at either 2 or 28 days was also, much greater in MSU than in Zebra (63, 26 vs 32, 9). The overall number of genes expressed at 28 days was 2.7 fold greater than at 2 days (63,32 vs 26,9). Overall, 49 genes or 14% of the total down-regulated genes showed expression that was conserved across genotypes and treatment durations. Thus down-regulation of gene expression is a predominant feature of bermudagrass crown tissues reaction to cold temperatures, especially in resistant MSU. This preponderance of down-regulated genes is most likely due to an extensive shut down of metabolic pathways during cold acclimation to a basal level of metabolism. Such a reduction was seen in sunflower where the large majority of genes were down regulated in response to chilling stress [42].

Overall expression was intensified in MSU vs Zebra as indicated in the bar chart in Fig 1B. The overall Log_2_ expression ratio of treated vs control averaged 1.88 (3.54-fold change) in MSU vs 1.45 (2.12 fold change) in Zebra (p value ≤ 0.001). Thus the intensity of expression is increased in cold resistant MSU. There was no significant changes in the overall level of expression between 2 and 28 day treated tissues nor was there a significant difference in the absolute value of expression for up-regulation compared to down regulated genes. Thus the predominant and significant difference in expression in this study was associated with genotype.

### Gene ontology (GO) functional analysis

The overall assessment using the slimmer functions reflect the major areas affected by cold temperatures. Of the 97 genes with functional NCBI matches, 56 showed GO functional annotations (29 up, 22 down and 5 mixed regulated). The few genes associated with GO functions in this study makes it difficult to construct an overall assessment of metabolic function in response to cold temperature. however there were a few patterns that deserve further discussion.

**Fig 2:**
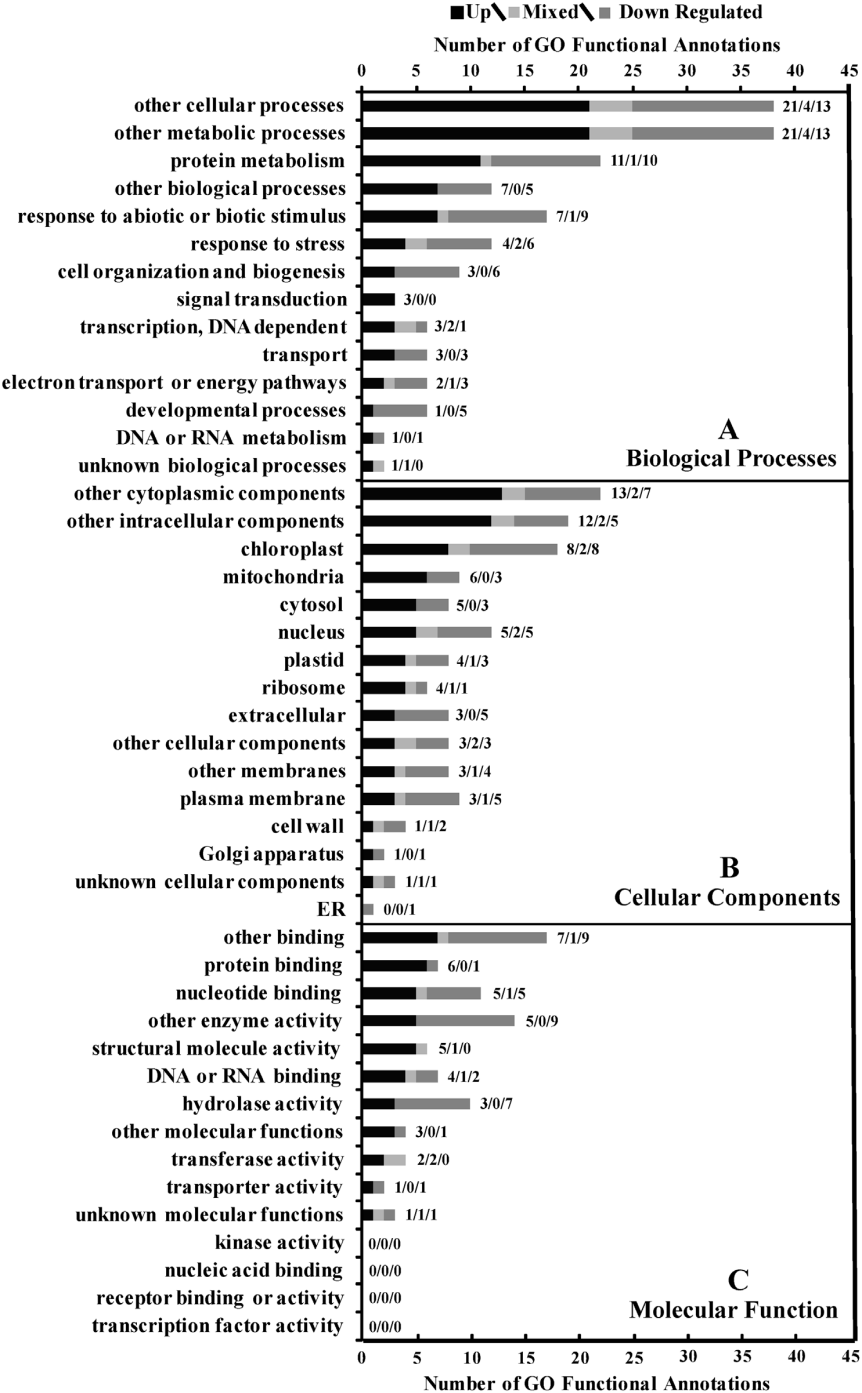
Overall GO functional analysis of bermudagrass gene expression under cold temperature conditions. **A** describes biological process domain, **B** cellular component domain and **C** molecular function domain, Sub domain descriptions are described on the y axis and numbers of genes with GO functions on the x axis. Data labels to the right of each bars reflect the up/mixed/or down numbers of regulated genes for each description.

Biological processes (Fig 2A) most affected by cold acclimation included: other cellular and other metabolic processes, protein metabolism, other biological processes and response to abiotic or biotic stimulus. All but three of the GO functionally annotated genes in this study were found associated with either the other cellular or the other metabolic processes. Other cellular processes exclude: signal transduction, cell organization, biogenesis and transport and may include cell cycle, cell adhesion, cell death etc. The other metabolic processes exclude protein, DNA, RNA, electron transport, and energy metabolism in addition to transcription, but may include lipid (not associated with energy), secondary metabolism, hormone, and carbohydrate metabolism (not associated with energy production). The other cellular and metabolic processes categories leaned towards up-regulation and developmental processes leaned heavily towards down-regulation. GO functional assignments of the primary biological processes such as energy, transport or transcription were much less represented in the GO functional analysis indicating that these primary growth processes were neither up nor down regulated under cold conditions. The fact that the other processes dominated the differential expression GO functional analysis, and that primary growth processes were much less apparent could be due to the adjustments associated with dormancy-like quiescent phase in response to cold temperatures.

Among the up-regulated biological processes that appeared to stand out were those associated with protein metabolism, protein folding, amino acid metabolism and translation. Over half of the up-regulated genes within this category have some association with these three protein functions and of these, translational processes were more prominent. Protein metabolism includes: translation, proteolysis, modifications, and folding. Here we found that a number of ribosomal proteins were up-regulated under cold temperature. Ribosomal proteins were thought to be associated with housekeeping genes and even used in microarray studies as expression controls. More recent investigations reveal that the ribosome translational apparatus is remodelled in response to a variety of stresses and environmental conditions [43]. Other areas of protein function that were up-regulated included amino acid metabolism with the up-regulation of specific genes associated with aspartate, phenylalanine, and branch chain amino acids biological processes. Protein modifications included DNAJ related proteins (protein folding), and polyubiquitin (protein targeting for degradation). Protein modifications are conceivably necessary to counteract the low temperature dependant changes and with respect to polyubiquitin in the recycling of nitrogen associated with protein turnover levels under cold conditions [44,45].

Other biological processes represent a collection of annotations that are found outside of all the other biological processes classifications schemes. Those other biological processes showing limited differential regulation in response to cold temperatures included: DNA dependent transcription, transport, electron transport or energy pathways, developmental processes DNA or RNA metabolism, and unknown biological processes.

In the biological processes, cell organization and biogenesis, and developmental processes were at least twice as likely to be down than up-regulated. Thus functions to maintain and expand the internal cellular organization and the developmental processes are likely curtailed and may be the result of the quiescent metabolic conditions in crown tissues exposed to prolonged cold temperatures.

Among the cellular components, other intracellular and other cytoplasmic were highly represented with cold acclimation genes (Fig 2B). Other intracellular domain includes functions related to protoplasm and nucleocytoplasm. Other cytoplasmic components exclude mitochondrion, plastid, ribosome, nucleus, cytosol (liquid phase of the cell excluding organelles), endoplasmic reticulum, and golgi apparatus, but including other subcellular structures. The chloroplast, nucleus and mitochondria categories were also highly represented in cold acclimating bermudagrass crown tissues. Other intracellular, other cytoplasmic, mitochondria, and ribosome domains contained nearly twice as many up- regulated genes as down- regulated genes, while extracellular plasma membrane categorized genes contained nearly twice as many down- regulated genes. Categories showing limited differential regulation included the cell wall, Golgi apparatus, unknown cellular component, and the endoplasmic reticulum.

Beside the “other” categories, genes associated with chloroplast function were most prominent. In bermudagrass crown tissues, the chloroplast actually reflects root plastids assignments. Like the chloroplasts, root plastids function in carbohydrate, amino acid, and fatty acid metabolism [46] and have been recently suggested to be associated with stress response [47]. Compared to chloroplasts, investigations of the root plastids have been relatively few in number even though a root plastid proteome was published recently [47]. In bermudagrass at least half of the plastid-associated categories were functionally associated with protein translation as discussed above with respect to biological processes categories.

Among the molecular function components (Fig 2C) other binding and other enzyme were highly represented, while nucleotide binding and hydrolase activity were moderately represented. Other binding excludes DNA, RNA, transcription factor, protein, and receptor binding/activity. Other enzyme category excludes kinase, hydrolase and transferase activities, but includes a wide range of enzyme activities with ligase, oxidoreductase, lyase, helicase, cyclase etc. Other enzyme activities and hydrolase activity were predominantly down- regulated in cold acclimating bermudagrass crowns. Number of hydrolase annotations were predominantly down-regulated and associated with protein degradation, or ATP catabolism or phosphorylation, possibly reflecting the low level of energy metabolism and signal transduction in quiescent tissues. Nucleotide binding includes interactions with nucleotides (DNA, and RNA) or nucleoside phosphates of ribose or deoxyribose (ATP, GTP etc.). Other molecular functions that were predominantly up-regulated included protein binding (non-covalent interactions with proteins), structural molecular activity (physical contribution to intra and intercellular macromolecular assemblies) and DNA or RNA binding (excludes transcription factor binding and is a subset of nucleotide binding indicated above). DNA or RNA binding annotations were predominantly those associated with ribosomal proteins and ATP synthase activities that were up- regulated (data not shown). Those molecular functions not showing associations with cold acclimating bermudagrass crowns included: transferase activities, transporter activity, unknown molecular functions, receptor binding or activity, transcription factor activity, kinase activity, nucleic acid binding (non-covalent interactions with nucleic acids- gene regulation), receptor binding or activity, and transcription factor activity.

#### Individual Gene Expression

Examining individual gene expression provides some clue as to functional changes in cold acclimated bermudagrass crowns. Individual genes varied in their level of expression with the greatest up-regulated gene showing 123-fold greater expression (log_2_X = 6.94) and the greatest down-regulated gene showing 41-fold reduced expression (log_2_X = -5.34) compared to control (Table 2A). The five most up-regulated genes overall included one known, a SAP-DIN1 (senescence associated protein-DIN1) and four genes of unknown function. Four out of five of these up-regulated genes previously showed differential response to spring dead spot disease (Table 2A). The five most down-regulated genes showed no similarity to sequences in the NCBI database, nor any previous differential response to spring dead spot (Table 2B).

**Table 2:** The highly differentially up and down-regulated genes in resistant MSU and susceptible Zebra crown tissues during cold acclimation treatment of 2 and 28 days.

NCBI Acc^a^	NCBI sequence match	E value	M2D^b^	M28D^c^	Z2D^d^	Z28D^e^	Fold change
**A**	**Up-regulated Genes**						
BQ826386	SAP- DIN1^f^	2.E-25	**6.94**	5.99	4.83	3.87	123
BQ826278	No significant similarity	Ns^g^	4.06	**5.46**	1.04	2.47	44
BG322365	No significant similarity	Ns^g^	3.28	**5.01**	0.90	1.62	32
DN986594	No significant similarity	Ns^g^	**4.86**	3.91	2.89	2.80	29
BQ826032	No significant similarity	Ns^g^	1.13	**4.24**	0.44	0.13	19
**B**	**Down-regulated Genes**						
DN987043	No significant similarity	Ns^g^	-3.39	**-5.34**	-0.59	-2.92	41
DN987053	No significant similarity	Ns^g^	-4.56	**-5.27**	-2.27	-3.22	28
DN987202	No significant similarity	Ns^g^	-4.88	**-5.14**	-2.72	-2.44	26
DN987203	No significant similarity	Ns^g^	-3.31	**-5.13**	-1.94	-2.71	26
DN986872	No significant similarity	Ns^g^	-3.78	**-4.84**	-1.93	-2.33	23

Bolded values represent maximum levels of expression

^a^NCBI Acc: Accession numbers starting with B were associated with spring dead spot library. Numbers with D were associated with the cold acclimation libraries.

^b^M2D- MSU 2 days cold acclimation treatment

^c^M28D- MSU 28 days cold acclimation treatment

^d^Z2D- Zebra 2 days cold acclimation treatment

^e^Z28D- Zebra 28 days cold acclimation treatment

^f^SAP DIN1- Senescence-associated protein DIN1

^g^Ns E value was not significant <0.001

Among those genes with NCBI sequence matches, the top five up-regulated genes included: senescence associated protein (SAP-DIN1), AAA type ATPase, sucrose synthase, acyl-CoA-binding protein and brassinoisteriod associated receptor kinase (Table 3A). Senescence associated gene in arabidopsis showed strong similarity to a dark induced (DIN) sulfide dehydrogenase and another dark induced protein (DIN), both being induced by dark and ABA treatments [48]. The AAA type ATPase is mechanistically involved in the energy dependent unfolding and disassembly of protein complexes associated with a diverse set of functions [49]. Sucrose synthase, a key enzyme that controls sucrose formation in sink tissues [50] was induced 8.6-fold in this study. Sucrose synthesis has been shown to be positively correlated with cold acclimation and tolerance [51]. This enzyme catalyses the formation of sucrose from UDP glucose and fructose and is reversible. Whither the enzyme functions in degradation or synthesis in bermudagrass crown tissues is not clear, but it is clear that the enzyme has controlling function in carbon partitioning in roots and may do so in bermudagrass crowns [52]. Earlier studies, dark induced genes have been associated with sucrose starvation [53]. However, Zhang showed that after 21 d of acclimation sucrose levels doubled [54], so sucrose starvation is an unlikely inducer with this particular gene. In previous studies with bermudagrass exposed to cold temperatures, sucrose levels increased 2-fold [54] which suggests a synthetic function. Sucrose may mediate some aspects of cold acclimation such as: compatible solute accumulation [54] to increase cellular osmotic potential and lowering freezing points, enhance membrane stabilization through membrane associations with phospholipids [55], or to reduce oxidative stress [56]. In arabidopsis there are six isozymes each with specific response to specific stresses including cold [57]. Furthermore, sucrose levels are also thought to regulate endo-dormancy in buds [58].

Acyl CoA binding proteins were shown to be highly up-regulated after 28 days of acclimation in tolerant but not susceptible genotypes. This is the first report of this protein being affected by cold temperatures. Acyl- CoA binding proteins transport Acyl CoA, from plastids to the endoplasmic reticulum. These proteins have a major effect on fatty acid composition, especially phospholipids [59]. Membrane lipid compositional changes in response to lower temperatures are a major determinant in tolerance to cold; therefore, acyl CoA binding proteins could be associated with changes in phospholipids in response to cold temperatures.

The five most down-regulated genes with NCBI matches included: universal stress protein (USP), dehydrin, glyoxylase I, ferrochelatase-2, and ribosomal protein S8 (Table 3B). The USP was down-regulated 11-fold. The USP constitute an ancient family of highly conserved proteins found in microorganisms and plants. Most are highly expressed in growth arrested cells, the exception being those involved in cold treatments where they are down-regulated, as in this study [60]. Dehydrins are a large family of proteins with unknown functions [61,62] that are generally up-regulated in response to dehydration, low temperature, and salt stress. Currently it is not known how they specifically function in cold acclimation [63]. In this study the dehydrin was found to be down- regulated 9-fold. The reason for this is somewhat puzzling, but dehydrins come as different isoforms whose expression may differ with different stresses and developmental stages [64]. Furthermore, not all dehydrins are induced by stress [65] and some are known to decrease during seed development [66]. Glyoxylase was down-regulated 8 fold after a 2 day exposure to cold in MSU but not after 28 days. This protein functions in detoxification pathways in bacteria and plants. Ferrochetelase is involved in heme biosynthesis in plants and the ribosomal protein S8 is part of the small ribosomal translational subunit involved in protein synthesis. The functional reason for the down-regulation of the latter three mentioned genes during cold treatments is unknown at this time.

**Table 3:** Genes with NCBI sequence similarities and the most up and down regulated in MSU and Zebra bermudagrass crown tissues when exposed to cold acclimation treatments.

NCBI Acc^a^	NCBI sequence match	E value	M2D^b^	M28D^c^	Z2D^d^	Z28D^e^	Fold change
**A**	**Up-regulated Genes**						
BQ826386	SAP- DIN1^f^	2.E-25	**6.94**	5.99	4.83	3.87	123
BG322297	AAA-type ATPase family protein	4.E-31	3.28	3.17	1.79	1.50	10
BQ826306	Sucrose synthase metabolism	5.E-71	3.11	2.93	2.14	1.34	9
DN987085	Aspartate aminotransferase	5.E-77	3.00	1.00	1.87	-0.32	8
BQ825934	Acyl-CoA-binding protein	2.E-21	0.82	2.78	0.48	0.57	7
**B**	**Down-regulated Genes**						
BQ826426	Universal stress protein	7.E-11	-2.32	-3.45	-1.74	-2.43	11
DN987480	Dehydrin DHN1	4.E-29	-3.15	-1.65	-0.38	-0.4	9
DN987028	Glyoxylase1	2.E-70	-3.05	-0.12	-2.05	0.15	8
DN987690	Ferrochelatase-2	9.E-28	-1.92	-2.76	-0.77	-1.86	7
DN987771	Ribosomal protein S8	4.E-18	-1.68	-2.37	-0.26	-1.66	5

Bolded values represent maximum level of expression

^a^ NCBI accession number: Numbers starting with B were associated with spring dead spot library, numbers starting with D associated with cold acclimation libraries

^b^ M2D: MSU 2 days cold acclimation treatment

^c^ M28D: MSU 28 days cold acclimation treatment

^d^ Z2D: Zebra 2 days cold acclimation treatment

^e^ Z28D: Zebra 28 days cold acclimation treatment

^f^ SAP DIN1: Senescence-associated protein DIN1

One of the main objectives of this work was to examine differences between cold resistant and susceptible backgrounds for differential gene expression in bermudagrass crowns with a particular interest in the MSU genetic background. Those genes that showed the greatest absolute-fold differential between MSU and Zebra lacked any similarity to genes in the NCBI database (Table 4). Five out of the ten were strongly up-regulated in MSU, four were strongly down-regulated, and one was strongly down-regulated in Zebra. Only two out of 10 were derived from SDS library

**Table 4:** Gene expression comparisons in resistant and susceptible backgrounds. Values are expressed as Log_2_ ratio of expression between cold treated and control tissues.

NCBI Acc^a^	NCBI sequence match	MSU Ave^b^	Zebra Ave^c^	ABS Value M-Z^d^	ABS Fold change^e^	Highest Expression^f^
DN986568	No homolog found	2.15	-0.89	3.04	8.22	MSU
BQ826278	No homolog found	4.76	1.76	3.00	8.01	MSU
DN987172	Hypothetical rice protein	-2.77	0.12	2.89	7.43	Zebra
DN985623	No homolog found	2.10	-0.79	2.89	7.41	MSU
BG322365	No homolog found	4.14	1.26	2.88	7.37	MSU
DN987003	No homolog found	-3.02	-0.15	2.87	7.33	Zebra
DN985637	No homolog found	2.29	-0.58	2.87	7.30	MSU
DN987206	Unknown rice protein	-4.00	-1.14	2.86	7.26	Zebra
DN988165	No homolog found	-2.68	0.14	2.82	7.08	Zebra
DN985763	No homolog found	0.23	-2.58	2.81	7.01	MSU

Bolded values represent maximum level of expression

^a^ NCBI accession number: Numbers starting with B were associated with spring dead spot library, numbers starting with D associated with cold acclimation libraries

^b^ MSU Ave: Average level of Log_2_ ratio for cultivar MSU between treated and control crown tissues averaged across time points

^c^ Zebra Ave: Average level of Log_2_ ratio for cultivar Zebra between treated and control crown tissues averaged across time points

^d^ ABS Value M-Z: The absolute value of the difference between cultivars MSU and Zebra in Log_2_ ratio of treated and control tissues

^e^ ABS Fold change: The absolute value of the fold change difference between MSU and Zebra treated and control tissues

^f^ Highest Expression: The cultivar MSU or Zebra with the highest level of expression

Average change in expression among cultivars were presented in Table 5 for those which has sequence annotations from NCBI database including: senescence associated protein in corn, AAA-type ATPase family protein in rice, sucrose synthase in rice, another senescence associated protein in the Easter lily, and an Acyl-CoA binding protein from ginseng. The difference ranged from 4.7 to 3.01-fold between MSU and Zebra. Those showing the greatest absolute difference in expression were aspartate amino transferase, RNA polymerase, and senescence associated protein DIN1. Aspartate amino transferase (AAT) was upregulated 9-fold in bermudagrass crowns. This enzyme functions in the synthesis or catabolism of the amino acids, aspartic acid and asparagine and is a key component in regulation of nitrogen metabolism [67]. There are no reports of its induction in plants in response to cold. This up-regulation contributes to the high GO functional activity in transferase and protein metabolisms in bermudagrass under cold acclimation conditions (Table 5). AAT may function in the slow recycling of nitrogen during cold conditions.

**Table 5:** Average change in gene expression among cultivars for top few genes. Values are expressed as Log_2_ ratio of expression between cold treated and control.

NCBI Acc^a^	NCBI sequence match	MSU Ave^b^	Zebra Ave^c^	ABS Value M-Z^d^	Fold change^e^	direction^f^
DN986555	Aspartate Aminotransferase	2.03	-0.20	2.24	4.71	Higher MSU
DN985609	RNA Polymerase	0.38	-1.75	2.13	4.39	Higher MSU
BQ826386	SAP DIN1^g^	6.47	4.35	2.12	4.34	Higher MSU
DN985666	Sucrose Synthase	-2.09	-0.19	1.90	3.72	Lower MSU
DN985574	Brahma-Associated Protein	-3.09	-1.26	1.82	3.54	Lower MSU
DN988913	Protein Kinase (AME2/AFC1)	-1.01	0.80	1.81	3.50	Lower MSU
DN986847	RabGAP/TBC Domain	1.23	-0.54	1.77	3.40	Higher MSU
DN987119	Ribosomal Protein S1	0.38	2.03	1.65	3.13	Lower MSU
DN987969	DnaJ-related Protein	-0.59	1.05	1.64	3.12	Lower MSU
DN987116	AAA-type ATPase	-3.29	-1.70	1.59	3.01	Lower MSU

Bolded values represent maximum level of expression

^a^ NCBI accession number: Numbers starting with B were associated with spring dead spot library, numbers starting with D associated with cold acclimation libraries

^b^ MSU Ave: Average level of Log_2_ ratio for cultivar MSU between treated and control crown tissues averaged across time points

^c^ Zebra Ave: Average level of Log_2_ ratio for cultivar Zebra between treated and control crown tissues averaged across time points

^d^ ABS Value M-Z: The absolute value of the difference between cultivars MSU and Zebra in Log_2_ ratio of treated and control tissues

^e^ ABS Fold change: The absolute value of the fold change difference between MSU and Zebra treated and control tissues

^f^ Highest Expression: The cultivar MSU or Zebra with the highest level of expression

^g^ SAP DIN1: Senescence-associated protein DIN1

The greatest absolute value differences in temporal gene expression between the 2 and 28 day acclimation periods among up-regulated genes averaged across resistance levels were determined for MSU only (Table 6). Of the top 10 showing differences, 8 were from genes with unknown NCBI matches. The exceptions were a virus polyprotein and Delta-1-Pyrroline-5-Carboxylate Synthetase gene, both of which were strongly down-regulated at 28 days and 2 days, respectively. Proteins that are synthesized into repeated segments and cleaved to make multiple proteins are called polyproteins. Viruses are known to make such proteins [68]. Recently antifreeze proteins that strongly mimic polyprotein structure were found in the Antarctic eel liver [69]. Cold hardy alfalfa variety up-regulated a retrotransposon gene with repetitive elements that was capable of insertion into a polyprotein like sequences [70]. However, the functional association of these retrotransposon sequences is unclear at this time. Normally DPCS genes are up-regulated in response to cold, salt and water stress [71,72] in metabolically active tissues in order to produce proline, a compatible solute. Here the gene was dramatically down-regulated in MSU crown tissues under cold conditions. This may be due to the down-regulation of normal metabolism in a dormancy-like condition where metabolic production of a compatible solute such as proline may be prohibitive.

**Table 6:** Differences in expression between 2 and 28 days (temporal difference) for MSU cultivar only.

NCBI Acc^a^	NCBI sequence match	MSU2^b^	MSU28^c^	abs value 28d-2d^d^	Fold Change	Direction^e^
DN987224	Virus Polyprotein	**-1.29**	**-3.98**	2.69	6.44	Down MSU2 &28
DN985664	No homolog found	**-0.61**	**-3.56**	2.95	7.75	Down MSU 28
DN987299	No homolog found	**-0.42**	**-3.43**	3.01	8.05	Down MSU 28
BG322345	DPCS^f^	**-3.05**	**-0.12**	3.14	8.81	Down MSU2
DN988458	No homolog found	**-1.15**	**-4.29**	3.14	8.81	Down MSU 2 &28
BQ826032	No homolog found	**1.13**	**4.24**	3.20	9.22	Up MSU 28
DN986612	No significant match	**-3.26**	**-0.06**	3.34	10.16	Down MSU2
DN986691	hypothetical protein	**3.22**	**-0.13**	3.34	10.16	Up MSU2
BQ826219	No homolog found	**-3.08**	**0.54**	3.62	12.29	Down MSU2
DN986664	No homolog found	**-2.94**	**1.12**	4.06	16.70	Down MSU2

^a^ NCBI accession number: Numbers starting with B were associated with spring dead spot library. Numbers starting with D associated with cold acclimation libraries

^b^ MSU2: Log2 ratio of treated vs control for MSU 2 day treatment

^c^ MSU28: Log2 ratio of treated vs control for MSU 28 day treatment

^d^ ABS value 28d-2d: Absolute value of the Log_2_ ratio of treated vs control difference between MSU 28 day and 2 day treatments

^e^ Direction: Direction in terms of greatest change between treated and control

^f^ DPCS: Delta-1-Pyrroline-5-Carboxylate Synthetase

### Validation of microarray experiment

**Fig 3:**
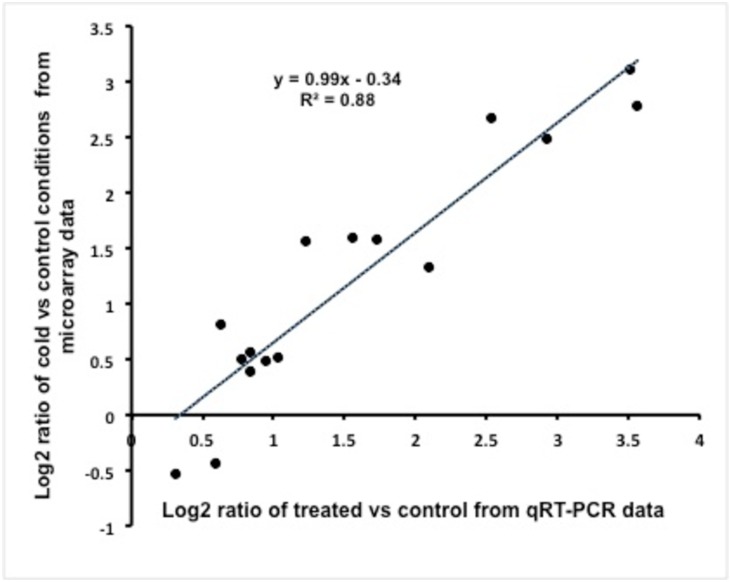
Scatter plot of microarray data represented as Log2 ratio of treated and control (y axis) and the Log 2 ΔΔC_T_ expression values for the q RT-PCR.

qRT-PCR validation of microarray expressions were confirmed for selected genes and treatments. Four genes were selected for evaluation across the two varieties and two time periods for semi-quantitative RT-PCR analysis (S3 Table). ΔΔC_T_ Values were transformed to log_2_ and compared with the microarray log _2_ ratios, which in turn were derived from cold acclimated and non-acclimated values from both varieties for a total of 16 validation points. Both the experiments were compared by scatter plot with the y-axis representing microarray data and the x-axis the qRT-PCR data (Fig 3). Correlation between log_2_ ratios of microarray and qRT-PCR ΔΔC_T_ expression values in cold acclimated and non-acclimated tissues revealed a very close relation with an R^2^ value of 0.88 (Fig 3). This close relationship strongly supports the validity of the microarray expression analysis.

## Conclusions

Low temperatures limit bermudagrass growth in North America. Research into the cold acclimation ability of non-cereal grasses provides a foundation to develop grasses capable of growing beyond their natural adaptation zones. There is limited literature available to help understand low temperature stress responses in bermudagrass, or other turf grasses. Our research is one of the first attempts to observe global gene expression changes in bermudagrass to low temperatures. We have developed a bermudagrass cold acclimated library by enriching genes for low temperature, and 3845 EST sequences were deposited in GenBank.

A surprising result was that a high percentage of the differentially expressed genes do not have sequence matches in public repositories, hence they are new contributions to the overall field of cold acclimation awaiting functional characterization. GO functional analysis reveals that these primary growth processes were neither up- nor down- regulated under cold conditions. The fact that the ‘other processes’ dominated the differential expression and that primary growth processes were much less apparent in the analysis could be a result of the tissue entering a quiescent phase in response to cold temperatures. Some of the genes that were induced by cold acclimation were involved in senescence, protein unfolding and disassembly, sucrose synthesis, membrane stability, osmotic potential, nitrogen metabolism and cytoskeletal mechanisms. Some of the repressed genes from this study were involved in embryogenesis, membrane protection and degradation of stored protein processes. Understanding qualitative and quantitative effects of these genes on the cold acclimation process of bermudagrass needs further study. Comparatively, more literature is available about cold acclimation responses for the above ground plant tissues than underground storage organ like bermudagrass crown tissues [73–76]. This study can complement the understanding of other warm season non-cereal grasses that are recently gaining importance. Differentially expressed genes from this study can be potential targets for developing molecular markers for cold tolerance which further can help plant breeders develop bermudagrass varieties that could be grown beyond the current adaptation zones.

## Supporting Information

S1 Fig Suppression Subtractive Hybridization (SSH) experimental design. (PDF)

S1 Table All the differentially expressed 586 unigenes from microarray analysis. Expression analysis was performed with low temperature treated samples verses control samples with in the genotype and ratios were transformed into log_2_ values.
(PDF)

S2 Table Differentially expressed genes, which have sequence similarities in the NCBI database. (PDF)

S3 Table RT-PCR analyses of selected cold resistant genes and their comparison with microarray expression. (PDF)
