# Supplementary material for: Transcriptional Analysis of Resistance to Low Temperatures in Bermudagrass Crown Tissues
Source: PLoS One. 2015 Sep 8;10(9):e0136433. doi: 10.1371/journal.pone.0136433 (PMC4562713; doi:10.1371/journal.pone.0136433)
Supplement: S1 Fig — (PDF) [file pone.0136433.s001.pdf]

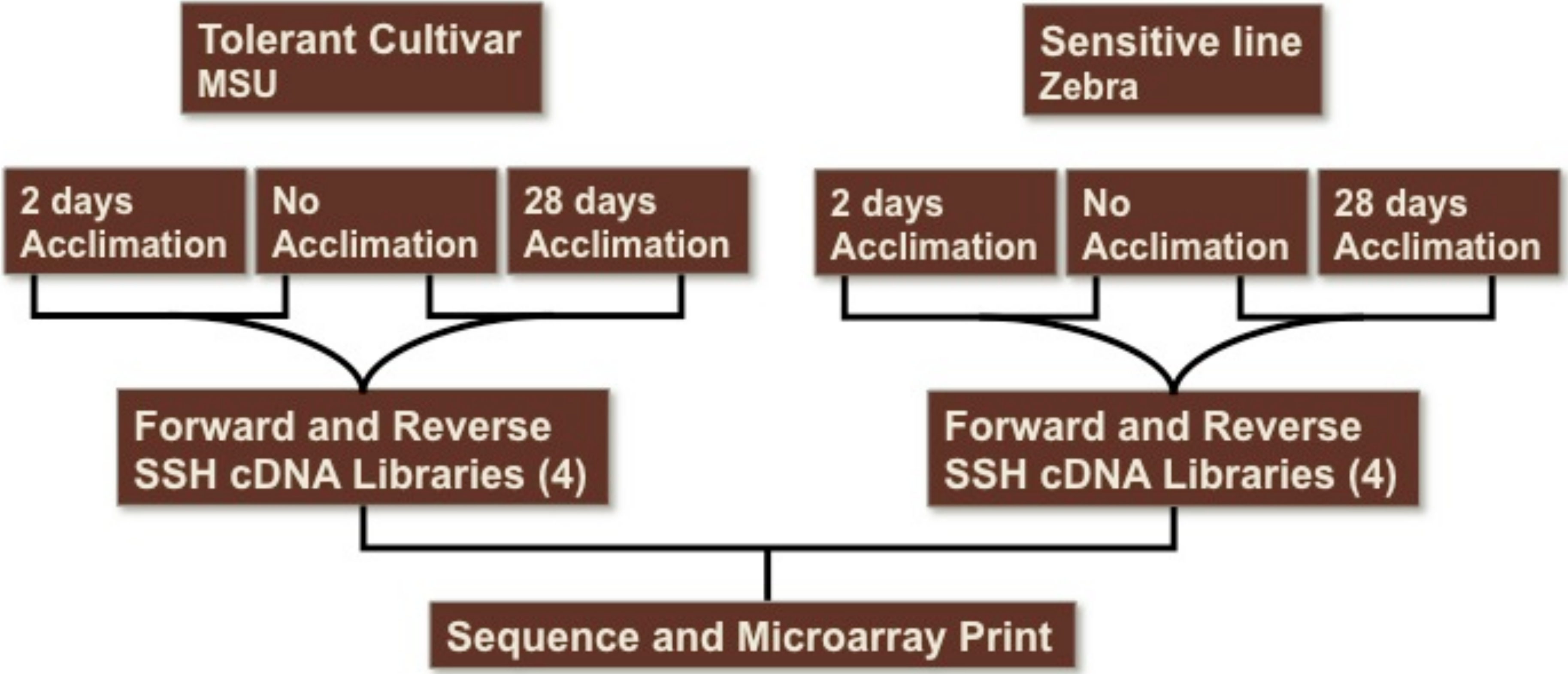
Tolerant Cultivar
MSU
Sensitive line
Zebra
2 days Acclimation
No Acclimation
28 days Acclimation
2 days Acclimation
No Acclimation
28 days Acclimation
Forward and Reverse SSH cDNA Libraries (4)
Forward and Reverse SSH cDNA Libraries (4)
Sequence and Microarray Print
