# Supplementary material for: Transcriptional Analysis of Resistance to Low Temperatures in Bermudagrass Crown Tissues
Source: PLoS One. 2015 Sep 8;10(9):e0136433. doi: 10.1371/journal.pone.0136433 (PMC4562713; doi:10.1371/journal.pone.0136433)
Supplement: S1 Table — Expression analysis was performed with low temperature treated samples verses control samples with in the genotype and ratios were transformed into log2 values. (PDF) [file pone.0136433.s002.pdf]

Acc #	Blast x Hits	E value	M2D	M28D	Z2D	Z28D
BQ826386	senescence-associated protein DIN1, Zea mays	2.00E-25	6.94	5.99	4.83	3.87
BQ826278	No significant similarity		4.06	5.46	1.04	2.47
BG322365	No significant similarity		3.28	5.01	0.90	1.62
BQ826032	No significant similarity		1.13	4.24	0.44	0.13
BQ826237	No significant similarity		1.34	4.20	1.30	2.25
DN986594	No significant similarity	5.60E-01	4.86	3.91	2.89	2.80
BQ826122	No significant similarity	3.40E+00	1.53	3.86	0.44	2.57
DN987451	No significant similarity	7.10E-01	3.35	3.74	3.56	3.29
BQ826236	No significant similarity	8.60E+00	1.61	3.73	0.58	1.57
BQ826295	hypothetical protein, Oryza sativa Indica	2.00E-18	3.99	3.65	3.32	2.36
BQ826235	No significant similarity		2.47	3.64	1.58	1.93
DN987383	No significant similarity	6.10E+00	3.95	3.62	3.31	3.26
DN986576	No significant similarity		3.87	3.51	2.31	2.32
DN988807	No significant similarity	5.50E-01	4.14	3.34	2.50	2.39
BQ826220	No significant similarity		1.29	3.21	0.91	2.04
BQ825991	No significant similarity	1.30E+00	2.51	3.21	1.23	1.58
BG322297	AAA-type ATPase family protein, Oryza sativa Japonica	4.00E-31	3.28	3.17	1.79	1.50
BQ826339	No significant similarity		3.01	3.13	0.96	1.71
BQ826250	No significant similarity		1.16	3.08	-0.46	0.29
DN988824	No significant similarity		0.60	3.08	-0.23	1.66
DN988554	No significant similarity		0.59	3.05	0.37	1.88
DN987645	No significant similarity	6.20E+00	3.30	2.96	3.17	3.11
BQ826049	No significant similarity	3.20E+00	3.51	2.94	1.48	1.23
BQ826287	AAA-type ATPase family protein, putative, expressed, Oryza sativa Japonica	2.00E-19	3.37	2.93	1.32	1.41
BQ826306	sucrose synthase , Oryza sativa Indica	5.00E-71	3.11	2.93	2.14	1.34
DN988441	No significant similarity	7.70E+00	1.05	2.92	0.21	0.44
BQ826030	No significant similarity	1.90E+00	0.98	2.90	0.29	0.54
BQ826269	No significant similarity	3.00E+00	2.20	2.88	1.54	1.57
BQ825965	No significant similarity	3.10E+00	1.59	2.84	0.82	2.76
BQ826150	No significant similarity	6.10E+00	0.75	2.84	0.01	1.07
BQ825934	Acyl-CoA-binding protein, Panax ginseng	2.00E-21	0.82	2.78	0.48	0.57
DN986572	No significant similarity	1.00E+00	2.47	2.73	1.28	1.38
BQ826073	No significant similarity	5.10E+00	1.15	2.65	0.65	0.36
DN985637	No significant similarity		1.94	2.65	-0.44	-0.71
DN985561	No significant similarity		2.66	2.64	2.09	1.96
DN987089	No significant similarity	2.00E+00	1.06	2.64	0.36	0.92
DN986321	No significant similarity		2.11	2.63	0.00	-0.71
DN986845	No significant similarity		2.40	2.56	-0.18	-0.29
BQ826261	No significant similarity		0.61	2.49	0.08	0.65
BQ826351	MAPK activating protein, Zea mays	2.00E-35	3.35	2.48	1.33	1.73
DN986579	No significant similarity		2.32	2.47	-0.39	-0.23
DN987367	hypothetical protein SORBIDRAFT_01g023641,Sorghum bicolor	3.00E-03	2.21	2.39	-0.03	-0.15
DN985623	hypothetical protein, Oryza sativa Indica	1.00E-24	1.83	2.37	-0.90	-0.69
BQ826017	No significant similarity		0.46	2.37	0.56	-0.33
DN986568	No significant similarity	2.70E-01	1.97	2.33	-0.42	-1.36
DN986700	No significant similarity	5.20E+00	2.22	2.29	-0.19	-0.67
DN986836	No significant similarity	2.60E+00	2.23	2.29	-0.19	-0.13
BQ826366	actin, Picea mariana	7.00E-19	2.21	2.28	1.37	1.64
BQ826323	No significant similarity		2.29	2.27	1.46	1.00
BQ825938	No significant similarity	5.90E+00	1.55	2.26	0.81	1.83
BQ826101	putative ADP-ribosylation factor, Pinus pinaster	3.00E-11	0.71	2.26	0.61	0.91
DN986723	No significant similarity		2.25	2.26	-0.29	-0.41
BG322336	GRAS family transcription factor, Populus trichocarpa	2.00E-96	2.15	2.22	1.65	1.37
BQ826273	No significant similarity		2.74	2.21	1.08	1.03
BQ826075	No significant similarity	1.10E+00	3.18	2.18	1.42	1.06
DN989177	No significant similarity		1.18	2.14	1.00	2.02
DN989174	ribosomal protein S1, Sorghum bicolor	7.00E-65	1.35	2.14	1.12	2.03
BQ826352	anthranilate phosphoribosyltransferase, Arabidopsis thaliana	2.00E-04	1.76	2.10	0.92	2.64
BQ826271	No significant similarity	4.00E+00	0.90	2.06	0.46	1.71
DN986682	No significant similarity	9.20E+00	1.71	2.03	2.24	3.47
DN986575	No significant similarity		1.89	1.98	-0.55	-0.39

Expression Level
5 to 7
3 to 5
1 to 3
-3 to -5
-1 to 1
-1 to -3
-3 to -5
-5 to -7

Acc #	Blast x Hits	E value	M2D	M28D	Z2D	Z28D	Expression Level
DN986891	No significant similarity		1.80	1.98	0.13	1.59	
DN986557	No significant similarity	1.60E+00	1.70	1.96	-0.61	-0.39	
BQ825943	ribosomal protein L11, Felis catus	2.00E-32	1.12	1.92	1.06	1.31	
	No significant similarity		-0.10	1.88	1.58	0.95	
DN986555	No significant similarity		2.19	1.88	-0.09	-0.32	
BQ826209	No significant similarity		0.22	1.87	-0.13	0.59	
DN986827	No significant similarity	1.00E-01	2.49	1.86	1.68	1.78	
BQ826336	No significant similarity	8.90E+00	1.15	1.79	1.68	1.89	
DN986903	No significant similarity		0.18	1.77	0.66	0.41	
BQ826214	No significant similarity		0.65	1.75	0.01	0.69	
DN986699	No significant similarity		1.46	1.75	-0.04	-0.14	
DN986846	No significant similarity	4.50E+00	1.76	1.73	-0.73	0.03	
DN986562	No significant similarity		2.34	1.72	-0.47	-1.06	
DN988455	No significant similarity	4.90E-01	1.90	1.70	0.54	1.27	
BQ826337	ribosomal protein S15, Triticum aestivum	3.00E-32	1.29	1.69	0.34	-0.08	
DN986632	No significant similarity		1.22	1.69	-0.48	-0.53	
BQ826040	No significant similarity		1.22	1.68	0.50	1.72	
DN988506	No significant similarity	7.70E+00	2.44	1.67	0.20	1.53	
DN985639	No significant similarity		1.10	1.66	-0.62	0.35	
DN986716	No significant similarity		1.63	1.64	1.38	0.90	
DN986635	No significant similarity		1.33	1.64	-0.38	-0.84	
DN986752	No significant similarity		-0.11	1.63	0.72	0.61	
DN986876	No significant similarity		1.14	1.62	-1.10	-1.08	
DN986571	No significant similarity		1.88	1.60	0.69	0.58	
	No significant similarity		0.88	1.60	-0.02	0.48	
BQ826252	No significant similarity	2.30E+00	0.47	1.59	0.46	0.81	
BQ826279	sucrose synthase, Sorghum bicolor	1.00E-81	1.59	1.59	0.52	-0.45	
DN986506	No significant similarity		1.61	1.58	-1.17	-0.97	
DN985640	No significant similarity		2.11	1.57	-0.54	-1.06	
DN986637	No significant similarity		0.69	1.57	0.21	-0.08	
BQ826356	putative protein kinase (AME2/AFC1), Oryza sativa Japonica	3.00E-43	0.39	1.56	-0.53	0.51	
BQ826072	hypothetical protein PTT, Pyrenophora teres	2.00E-10	0.10	1.54	-0.76	1.01	
DN988689	No significant similarity		1.11	1.54	-0.66	0.32	
DN985687	No significant similarity		1.54	1.48	-0.06	-0.43	
BQ826239	beta-1,3-glucanase, Oryza sativa	1.00E-08	0.70	1.47	0.61	1.56	
DN988735	No significant similarity		1.13	1.47	1.42	1.52	
BQ826207	No significant similarity		1.90	1.41	-0.11	1.05	
DN987459	dehydrin 13, Zea mays	4.00E-06	-0.39	1.40	0.10	1.87	
DN986561	No significant similarity		1.03	1.38	0.26	-0.19	
DN988712	No significant similarity		1.03	1.36	-0.20	-0.24	
DN986805	No significant similarity	1.90E-01	0.61	1.35	-0.45	0.41	
DN988500	No significant similarity		1.60	1.33	0.75	0.53	
DN985926	hypothetical protein SORBIDRAFT_03g034090, Sorghum bicolor	7.00E-17	1.46	1.33	0.83	0.18	
BQ826005	No significant similarity		1.17	1.32	0.20	0.87	
DN986552	No significant similarity		1.50	1.31	-0.08	1.19	
DN988544	unknown, Zea mays	7.00E-06	0.58	1.31	0.63	-0.11	
DN988922	RNA polymerase beta' subunit, Sorghum bicolor	4.00E-13	0.95	1.30	-0.65	-1.03	
DN986218	No significant similarity		1.12	1.30	-0.05	-0.75	
DN987131	No significant similarity		1.00	1.28	2.12	3.21	
DN986847	No significant similarity	1.70E+00	1.18	1.28	0.11	-1.19	
BG322363	No significant similarity		1.56	1.27	0.51	0.58	
DN986835	No significant similarity	8.90E+00	1.27	1.26	0.02	-1.03	
BQ826232	No significant similarity	3.90E+00	1.18	1.26	-2.17	0.63	
DN988980	ferrochelatase-2, Zea mays	1.00E-06	0.91	1.25	0.25	0.69	
DN988344	putative senescence-associated protein, Lilium longiflorum	3.00E-11	1.11	1.24	0.86	1.18	
BQ826385	40S ribosomal protein S11, Zea mays	2.00E-80	0.82	1.20	0.82	1.00	
DN987102	ketol-acid reductoisomerase, Zea mays	3.00E-74	0.47	1.20	1.10	0.78	
DN985383	No significant similarity		0.31	1.18	0.20	1.19	
DN987944	gibberellin 20 oxidase 2, Oryza sativa Indica	9.00E-03	1.89	1.17	0.82	1.07	
DN986856	No significant similarity	3.70E-01	1.54	1.17	-0.33	-0.41	
DN986649	No significant similarity	8.50E+00	1.08	1.15	0.44	0.05	
DN988370	putative senescence-associated protein, Pisum sativum	2.00E-25	1.33	1.15	1.04	1.21	

Acc #	Blast x Hits	E value	M2D	M28D	Z2D	Z28D
DN986786	No significant similarity	8.40E+00	0.56	1.13	0.85	0.74
DN988507	No significant similarity		1.22	1.13	1.21	1.30
DN986664	No significant similarity		-2.94	1.12	0.39	0.13
BG322337	No significant similarity		0.42	1.12	0.52	1.14
DN987134	No significant similarity	3.20E+00	0.93	1.12	0.25	0.70
DN987924	bHLH transcription factor-like protein, Oryza sativa Japonica	2.00E-06	1.07	1.10	0.80	0.51
DN986854	No significant similarity	7.10E+00	1.01	1.10	-0.17	-1.61
BQ825958	putative microtubule-associated protein MAP65-1a, (	1.00E-18	1.52	1.10	1.45	0.75
BQ826358	hypothetical protein Patl_3017 [Pseudoalteromonas atlantica T6c] >gb|ABG41525.1| protein of unknown function DUF482 [Pseudoalteromonas atlantica T6c]	1.40E+00	1.44	1.10	1.16	-0.40
DN986512	No significant similarity	2.10E+00	0.12	1.10	-0.35	0.00
BG322354	elongation factor 1-gamma 3, Oryza sativa Indica	1.00E-22	0.46	1.09	0.38	1.06
DN986650	No significant similarity		1.35	1.09	-0.28	-0.02
DN986247	No significant similarity		0.65	1.09	0.79	1.16
DN989089	BRASSINOSTEROID INSENSITIVE 1-associated receptor kinase 1, Zea mays	2.00E-54	0.70	1.09	0.10	0.46
BQ826357	phenylalanine ammonia-lyase, Zea mays	6.00E-17	0.47	1.08	0.66	1.36
DN987387	hypothetical protein SORBIDRAFT_0531s002010, Sorghum bicolor	1.00E-11	0.17	1.08	0.94	1.25
DN988946	No significant similarity	5.40E+00	1.00	1.08	0.65	0.59
DN988725	No significant similarity	8.60E+00	1.01	1.08	1.54	0.65
BQ825936	putative nucleolar GTP-binding protein, Oryza sativa Japonica Group	2.00E-21	0.51	1.08	0.60	1.34
BQ826228	No significant similarity		0.05	1.07	0.62	-0.81
DN986840	No significant similarity	6.90E-02	1.43	1.06	0.08	0.12
DN986648	No significant similarity	9.10E-01	1.16	1.06	-0.02	0.74
DN986701	No significant similarity	6.50E+00	0.72	1.06	0.00	0.71
DN988728	No significant similarity	3.90E+00	0.68	1.06	0.34	0.46
DN988463	No significant similarity		0.93	1.05	0.87	1.12
DN986908	No significant similarity		-1.64	1.05	-2.60	-0.34
BQ826267	60S ribosomal protein L37, Zea mays	1.00E-07	0.90	1.04	0.28	-0.08
DN985559	No significant similarity		1.39	1.03	-0.33	-0.80
DN987168	No significant similarity		1.76	1.03	0.65	0.83
DN987087	No significant similarity	3.80E+00	2.13	1.03	2.04	1.63
DN986610	No significant similarity	5.20E+00	1.42	1.02	-0.62	-0.26
BQ825969	expressed protein, Oryza sativa Japonica	1.00E-42	0.87	1.01	0.06	0.86
DN986539	No significant similarity	2.90E-01	0.04	1.01	0.49	1.40
DN987195	No significant similarity		1.19	1.01	-0.63	-1.06
DN987085	aspartate aminotransferase, Triticum aestivum	5.00E-77	3.00	1.00	1.87	-0.32
DN988556	No significant similarity		0.61	0.98	1.08	1.26
DN986530	No significant similarity	7.10E+00	0.44	0.98	1.35	1.59
DN988448	No significant similarity		-1.60	0.98	-0.58	1.02
DN987809	putative senescence-associated protein, Lilium longiflorum	5.00E-41	0.75	0.98	0.62	1.19
DN986683	No significant similarity	6.50E+00	1.55	0.97	0.32	1.03
BG322316	No significant similarity		0.50	0.96	0.90	1.39
DN987271	No significant similarity	9.80E+00	0.58	0.96	0.75	-1.01
BG322335	No significant similarity		1.26	0.95	1.24	1.11
BQ826205	No significant similarity		1.17	0.93	0.55	0.71
DN986559	No significant similarity	4.70E-01	0.93	0.93	-0.33	-1.53
DN987620	putative senescence-associated protein, Lilium longiflorum	1.00E-15	0.88	0.93	0.92	1.29
DN986790	No significant similarity	1.20E+00	1.46	0.92	0.69	-1.09
DN988861	No significant similarity	7.10E+00	-1.01	0.88	-1.01	0.57
DN987426	No significant similarity	3.90E+00	0.23	0.87	-0.09	1.35
BQ826091	No significant similarity		0.31	0.87	0.17	1.59
DN988543	von Willebrand factor type A domain containing protein, expressed, Oryza sativa Japonica	2.00E-72	1.97	0.86	-0.12	0.18

Expression Level

Acc #	Blast x Hits	E value	M2D	M28D	Z2D	Z28D	Expression Level
DN986141	hypothetical protein SORBIDRAFT_0057s002150, Sorghum bicolor	7.00E-60	1.10	0.86	-0.18	-0.41	
DN987056	No significant similarity	2.20E+00	2.07	0.86	1.47	0.54	
BQ826270	No significant similarity		-0.06	0.83	0.28	2.33	
DN986678	No significant similarity		1.19	0.82	1.16	1.54	
DN986689	No significant similarity		0.70	0.81	-0.33	-1.52	
DN988656	No significant similarity	4.70E+00	0.65	0.80	1.14	1.62	
DN986525	No significant similarity		0.75	0.79	1.26	0.99	
BQ826445	LIM domain protein GLIM1a, Populus tremula x Populus alba	1.00E-60	0.09	0.79	1.12	1.40	
DN987083	No significant similarity	1.00E+01	0.50	0.78	1.41	1.37	
DN986600	No significant similarity		-0.15	0.78	-0.02	-2.20	
DN988443	No significant similarity		-0.21	0.77	-0.96	1.61	
DN988570	No significant similarity		-0.03	0.77	0.08	1.37	
DN986510	No significant similarity		1.37	0.76	0.14	-0.07	
DN988446	No significant similarity	1.40E+00	1.03	0.76	0.34	1.14	
DN986445	polyprotein, Bermuda grass mosaic virus	3.00E-10	0.93	0.75	-0.17	-1.46	
BQ825962	No significant similarity	2.50E+00	-0.35	0.75	-0.42	1.47	
DN987119	No significant similarity	1.60E-01	0.03	0.73	1.75	2.30	
BQ826428	No significant similarity		1.11	0.73	1.49	0.91	
DN989054	senescence-associated protein, Picea abies	1.00E-68	0.62	0.71	0.73	1.01	
DN986688	No significant similarity	3.10E+00	2.14	0.70	0.97	0.21	
DN987196	Elastin binding protein EbpS, Staphylococcus lugdunensis HKU09-01	1.00E-03	0.68	0.68	-1.74	-2.34	
DN986599	No significant similarity	1.00E+00	1.30	0.67	0.18	1.10	
DN987779	putative senescence-associated protein, Lilium longiflorum	1.00E-50	0.15	0.66	-0.10	1.02	
BQ826096	No significant similarity		0.66	0.65	0.90	2.12	
DN987061	No significant similarity		0.82	0.64	1.49	1.01	
DN987187	No significant similarity		0.29	0.62	-1.81	-2.35	
DN986596	No significant similarity		2.14	0.62	0.06	0.25	
DN988527	No significant similarity		0.62	0.61	0.57	1.02	
DN987204	No significant similarity	2.30E+00	-1.33	0.58	-1.34	0.51	
DN987062	No significant similarity		0.17	0.58	0.52	1.24	
BQ826241	No significant similarity	8.70E-01	1.42	0.55	0.90	0.81	
BQ826329	ATP synthase subunit 6, Phaeosphaeria nodorum SN15	2.00E-12	-0.01	0.55	0.69	2.22	
BQ826221	No significant similarity	1.10E-01	-0.97	0.54	-0.69	1.25	
BQ826219	No significant similarity	1.00E+00	-3.08	0.54	1.51	0.59	
DN988529	No significant similarity	4.30E-01	-1.00	0.51	0.80	1.25	
BQ825985	No significant similarity	2.00E-03	0.35	0.51	0.41	1.71	
DN986567	No significant similarity		-1.17	0.50	-1.56	0.22	
BQ826262	hypothetical protein SORBIDRAFT_02g027230, Sorghum bicolor	6.00E-20	1.10	0.50	1.40	0.75	
BQ826268	No significant similarity		0.72	0.47	-0.18	2.14	
DN987448	Auxin-induced in root cultures protein 12 precursor, putative, Ricinus communis	4.00E-05	-0.79	0.44	-0.11	1.04	
DN986589	No significant similarity		1.10	0.44	0.44	-1.19	
DN986707	No significant similarity	5.80E+00	-1.97	0.44	-1.38	0.05	
DN987932	CBL-interacting serine/threonine-protein kinase 1, Zea mays	2.00E-04	1.40	0.41	-0.14	0.17	
BQ826234	No significant similarity		0.35	0.38	0.78	2.28	
DN987410	No significant similarity	1.10E+00	-0.66	0.37	1.48	1.79	
DN986448	No significant similarity		1.34	0.37	0.26	-2.31	
BQ825981	No significant similarity	2.20E+00	-1.36	0.37	-0.50	1.09	
DN986976	No significant similarity	8.80E+00	1.59	0.35	1.74	1.24	
DN985609	No significant similarity		0.43	0.33	-0.60	-2.90	
BQ826001	No significant similarity	6.70E+00	0.27	0.33	0.83	1.76	
BG322307	RabGAP/TBC domain-containing protein, Arabidopsis thaliana	2.00E-33	1.61	0.30	0.78	0.53	
DN988485	No significant similarity		0.43	0.30	1.06	0.46	
DN986850	No significant similarity	3.20E+00	1.22	0.30	-0.54	-0.29	
BQ825927	No significant similarity	6.40E-02	0.74	0.29	0.87	1.23	
DN988809	No significant similarity	5.20E+00	0.28	0.28	0.67	1.13	
DN986607	No significant similarity		0.27	0.28	-0.04	-2.20	
DN985606	xylanase inhibitor protein 1, Zea mays	7.00E-13	0.84	0.27	0.45	-1.25	

Acc #	Blast x Hits	E value	M2D	M28D	Z2D	Z28D	Expression Level
DN988667	No significant similarity	2.50E+00	-1.22	0.26	-1.62	-0.16	
DN987274	cation transport protein chaC, Zea mays	3.00E-25	0.74	0.26	0.92	1.11	
DN986592	No significant similarity	3.70E+00	-0.71	0.26	-1.04	0.42	
DN986703	Brahma-associated protein 111kD, Drosophila melanogaster	8.00E-04	-0.76	0.26	-2.91	-1.25	
BQ826344	ubiquitin-like protein, Triticum aestivum	1.00E-14	-0.22	0.24	0.99	1.58	
DN987070	No significant similarity		-1.23	0.24	-1.79	-0.67	
DN986642	No significant similarity		0.48	0.24	1.38	0.18	
DN986642	No significant similarity		0.48	0.22	-0.07	-2.12	
DN988555	No significant similarity		-1.01	0.22	0.58	1.34	
DN988333	putative senescence-associated protein, Pyrus communis	5.00E-16	0.15	0.20	0.12	1.21	
DN986663	No significant similarity	2.20E+00	-2.49	0.19	-1.99	0.03	
DN987058	CBL-interacting protein kinase 25, Sorghum bicolor	6.00E-19	-0.49	0.19	-1.03	0.24	
DN988856	No significant similarity		-0.09	0.16	-0.50	1.58	
BG322364	No significant similarity	1.60E+00	0.15	0.14	-1.02	-0.09	
DN985763	No significant similarity		0.31	0.14	-2.39	-2.77	
DN986331	No significant similarity		-0.05	0.11	-1.64	-2.24	
DN986838	No significant similarity	7.70E-01	0.47	0.11	0.30	-1.27	
BQ826412	DnaJ protein, putative, Oryza sativa Japonica	1.00E-56	0.19	0.10	1.14	0.00	
DN986676	No significant similarity		-0.95	0.09	-2.37	-0.49	
DN988840	No significant similarity		-1.14	0.09	-3.19	-0.81	
DN987408	hypothetical protein CJBH_1340, Campylobacter jejuni	2.00E-06	0.26	0.08	1.04	1.47	
DN988112	No significant similarity	4.20E+00	-2.66	0.07	-1.49	0.45	
DN986704	No significant similarity	7.10E+00	-0.80	0.06	0.89	1.23	
DN986601	No significant similarity		-2.26	0.06	-2.24	-0.14	
DN988498	No significant similarity		-1.96	0.05	-0.66	0.74	
DN986702	No significant similarity	1.10E+00	-1.40	0.04	-0.60	-0.72	
DN987174	No significant similarity	6.90E-01	-0.76	0.03	-1.31	-0.07	
BQ826254	No significant similarity		0.30	-0.02	0.90	1.51	
DN986554	No significant similarity		0.35	-0.03	0.11	-2.14	
DN986612	No significant similarity		-3.26	-0.06	-2.06	0.10	
DN987352	PREDICTED: inverted formin-2, Pan troglodytes	8.00E-05	-1.53	-0.07	0.08	0.57	
DN985630	No significant similarity		-2.87	-0.07	-2.56	-0.16	
BQ826404	No significant similarity		0.25	-0.08	1.27	0.64	
DN986866	No significant similarity	2.80E-02	-1.21	-0.09	-0.21	-0.59	
DN986611	No significant similarity	3.90E+00	-1.14	-0.09	-1.78	0.51	
DN986839	No significant similarity		0.39	-0.12	0.47	-1.99	
BG322345	Ubiquitin-conjugating enzyme family protein, Oryza sativa Japonica	2.00E-70	-3.05	-0.12	-2.05	0.15	
DN986691	No significant similarity	2.50E+00	3.22	-0.13	2.00	0.92	
BG322311	ascorbate peroxidase, Hordeum vulgare	1.00E-39	-1.39	-0.13	-0.36	-0.05	
DN985625	No significant similarity	3.70E+00	-3.01	-0.14	-2.15	0.00	
DN986843	No significant similarity	4.30E-01	0.14	-0.16	-0.28	-1.80	
BQ826409	No significant similarity		0.64	-0.17	1.17	0.24	
DN985676	No significant similarity		-2.71	-0.18	0.21	-0.79	
DN986516	No significant similarity		-1.19	-0.18	-1.69	-0.08	
DN988483	CTP synthase, Zea mays	1.00E-18	-0.40	-0.19	1.04	1.36	
DN987486	No significant similarity		-0.05	-0.19	1.14	-1.07	
DN986634	No significant similarity		-1.16	-0.20	-1.72	-0.65	
BQ826421	No significant similarity		-0.47	-0.21	1.16	1.07	
DN987650	ribosomal protein S8, Oryza sativa Japonica	3.00E-68	-0.16	-0.21	1.44	0.60	
DN987215	No significant similarity		-0.33	-0.22	-0.31	-1.01	
DN987474	Adenosylhomocysteinase, putative, expressed, Oryza sativa Japonica	2.00E-33	-1.29	-0.23	-0.38	-0.28	
DN986134	No significant similarity	8.20E+00	-1.97	-0.24	-1.48	-1.84	
DN987285	No significant similarity		1.16	-0.24	1.34	1.00	
DN985543	No significant similarity	4.30E-01	0.21	-0.25	-0.11	-1.92	
DN988883	No significant similarity		-2.41	-0.26	-1.93	0.08	
BQ826076	No significant similarity	5.70E+00	-2.33	-0.27	-2.15	0.19	
DN986826	No significant similarity		-1.66	-0.29	-1.07	-1.26	
DN987000	No significant similarity		-0.13	-0.31	1.65	0.41	
DN986013	No significant similarity	6.20E+00	-1.00	-0.31	-0.75	-0.70	
BQ825966	No significant similarity	3.10E+00	0.36	-0.33	1.02	0.30	

Acc #	Blast x Hits	E value	M2D	M28D	Z2D	Z28D	Expression Level
DN988670	No significant similarity		0.64	-0.34	1.20	1.28	
DN985458	putative cysteine proteinase, Hordeum vulgare	1.00E-11	-0.99	-0.34	-1.05	-0.72	
DN987494	NADP-dependent malic enzyme, Zea mays	2.00E-06	-1.32	-0.34	-1.24	-0.75	
DN987399	elongation factor 1-alpha, Zea mays	9.00E-81	-1.49	-0.35	0.63	1.09	
DN987182	No significant similarity	6.60E+00	-0.13	-0.36	1.14	-1.06	
DN987282	No significant similarity		-0.17	-0.37	-0.44	-1.13	
DN985401	No significant similarity		-1.23	-0.39	-1.54	-1.25	
DN987770	No significant similarity	1.60E+00	0.00	-0.39	0.87	1.13	
DN985397	ribosomal protein S3, Leersia tisserantii	9.00E-29	0.33	-0.39	1.68	0.64	
DN987589	No significant similarity		0.14	-0.41	-1.77	-1.49	
DN988070	No significant similarity		-1.37	-0.41	-1.10	-0.59	
DN988913	S-adenosylmethionine synthetase, Oryza sativa Indica	2.00E-72	-1.59	-0.42	0.50	1.10	
DN988906	hypothetical protein SORBIDRAFT_03g005610, Sorghum bicolor	2.00E-10	-0.09	-0.43	0.79	1.09	
DN988747	No significant similarity	2.60E-01	0.65	-0.44	1.61	1.28	
DN986792	No significant similarity		0.30	-0.48	-0.06	-1.04	
DN985991	No significant similarity	9.60E+00	-1.18	-0.49	-0.93	-0.43	
BG322357	F-box domain protein, Zea mays	5.00E-20	-0.36	-0.51	0.68	1.55	
DN988848	No significant similarity	8.40E+00	-1.41	-0.53	-2.27	-0.11	
DN987135	No significant similarity	5.10E-01	-1.41	-0.53	-1.63	-0.33	
DN987357	No significant similarity		-0.60	-0.54	1.00	-1.02	
DN986641	No significant similarity	8.60E-01	-0.86	-0.54	-1.28	-1.03	
DN988503	No significant similarity		-1.44	-0.55	-0.19	-0.61	
DN986958	No significant similarity		-0.06	-0.59	1.42	0.51	
DN986653	No significant similarity		-3.28	-0.60	-2.43	-0.24	
DN985777	No significant similarity		-1.99	-0.62	-1.26	-0.30	
DN987251	No significant similarity	9.70E-01	0.28	-0.65	1.28	0.86	
DN987179	MIP26658p, Drosophila melanogaster	9.00E-03	-0.46	-0.65	-0.89	-1.28	
DN987897	aspartic proteinase oryzasin-1, Zea mays	5.00E-53	-1.07	-0.66	-0.25	-0.95	
DN985617	No significant similarity		-1.77	-0.69	-1.33	-2.30	
DN986665	No significant similarity	6.30E-02	-1.74	-0.70	-0.92	-0.25	
DN986965	No significant similarity	1.50E+00	-1.45	-0.72	-1.70	-1.41	
DN988142	No significant similarity		-1.11	-0.74	-0.29	-1.11	
DN988920	autophagy-related protein 8 precursor, Zea mays	2.00E-37	-1.14	-0.75	-0.35	-0.76	
DN987097	No significant similarity		-1.28	-0.76	-1.43	-1.53	
DN987115	No significant similarity		-1.63	-0.78	-0.30	0.48	
BQ826382	No significant similarity	2.40E+00	-1.05	-0.78	0.28	0.16	
BQ826052	No significant similarity	2.40E+00	-2.65	-0.79	-2.54	-0.19	
DN987136	No significant similarity		-1.15	-0.79	-0.35	-1.19	
DN987054	No significant similarity		-0.82	-0.84	-1.76	-1.96	
DN987404	No significant similarity		-1.57	-0.86	-0.01	-0.40	
DN985437	No significant similarity		-2.72	-0.87	-1.47	-0.37	
DN985444	No significant similarity		-1.71	-0.87	-1.56	-0.41	
DN988453	Putative phosphoinositide phosphatase, Oryza sativa Japonica Group	1.00E-68	-0.69	-0.88	-1.26	-0.38	
DN985816	No significant similarity		-0.54	-0.89	-0.65	-1.14	
DN986868	No significant similarity		-1.74	-0.90	-0.27	-0.55	
DN986511	No significant similarity	1.60E+00	-2.30	-0.90	-1.57	-0.21	
DN986971	cysteine proteinase 1 precursor, Zea mays	7.00E-90	-1.63	-0.90	-0.40	-0.27	
DN988488	hypothetical protein ECDG_04665, Escherichia coli B185	4.00E-06	-0.40	-0.92	-3.03	-1.11	
DN988776	No significant similarity		-0.82	-0.93	1.29	0.19	
DN988559	No significant similarity	8.40E-02	1.08	-0.94	0.72	-0.63	
DN988519	No significant similarity		-0.05	-0.94	-2.53	-1.15	
DN987146	No significant similarity	9.00E+00	1.58	-0.95	0.78	0.55	
DN988843	No significant similarity		-0.42	-0.95	-0.14	1.11	
DN988512	putative CorA-like Mg2+ transporter protein, Oryza sativa Japonica	4.00E-27	-1.70	-0.97	-1.25	-0.14	
DN988610	No significant similarity		-1.62	-0.99	-0.42	-0.21	
DN987135	No significant similarity	5.10E-01	-1.41	-1.02	-1.40	-1.04	
DN986977	arginyl-tRNA synthetase, Zea mays	5.00E-84	-0.95	-1.02	0.03	-1.19	
DN987429	No significant similarity	3.80E-01	-1.46	-1.03	-1.06	-0.36	

Acc #	Blast x Hits	E value	M2D	M28D	Z2D	Z28D	Expression Level
DN988922	RNA polymerase beta' subunit, Sorghum bicolor	4.00E-13	0.93	-1.03	1.68	-0.10	
DN988764	No significant similarity	6.40E+00	0.01	-1.03	0.24	-0.58	
DN987065	MtN19-like protein, Arabidopsis thaliana	1.00E-52	-0.42	-1.04	-0.33	-0.52	
DN988817	Hypothetical protein COLAER_01671, Collinsella aerofaciens	1.00E-34	-0.65	-1.04	-0.63	-0.59	
DN986646	No significant similarity		-0.47	-1.05	-0.87	-0.63	
DN986584	No significant similarity		-0.72	-1.05	0.41	-0.54	
DN987035	No significant similarity	3.80E-01	-2.24	-1.05	-0.77	-1.00	
DN987048	No significant similarity		-0.84	-1.05	-1.00	-0.94	
DN987355	No significant similarity	3.20E+00	-0.86	-1.05	-1.12	-1.52	
DN987024	No significant similarity	1.30E+00	-0.25	-1.06	-2.36	-0.98	
DN986615	No significant similarity	3.80E+00	-0.91	-1.06	-0.44	-0.43	
DN988602	No significant similarity		-0.73	-1.06	0.57	0.12	
BQ826294	acyl carrier protein, Zea	2.00E-03	-0.83	-1.07	0.19	0.26	
DN989159	hypothetical protein SORBIDRAFT_0057s002150, Sorghum bicolor	2.00E-60	0.40	-1.07	-0.04	0.19	
DN987167	No significant similarity	3.10E+00	-0.56	-1.10	-0.50	-0.87	
DN986645	No significant similarity		-1.09	-1.12	-1.19	-1.14	
DN987246	No significant similarity	6.00E-02	-0.48	-1.12	0.20	-0.77	
DN985542	No significant similarity	2.80E+00	-1.40	-1.13	-1.01	-0.97	
DN985396	conserved hypothetical protein, Clostridium butyricum	2.00E-04	-0.16	-1.14	-0.27	-0.51	
DN985524	delta 1-pyrroline-5-carboxylate synthetase 1, Sorghum bicolor	6.00E-21	-1.84	-1.14	-1.86	-1.92	
DN987137	No significant similarity	7.20E-01	-2.20	-1.14	-0.48	-1.04	
DN987160	No significant similarity		-0.78	-1.15	-0.72	-1.43	
DN987101	No significant similarity		-0.09	-1.15	-0.64	-1.19	
DN988444	No significant similarity	9.40E+00	-0.07	-1.15	0.47	-0.84	
DN988601	No significant similarity		-0.62	-1.16	0.74	0.19	
DN986622	No significant similarity		-0.13	-1.16	-0.12	-0.38	
DN986698	No significant similarity	4.50E+00	-0.46	-1.17	-1.10	-1.19	
DN988415	No significant similarity		-0.74	-1.17	0.63	-0.68	
DN985523	No significant similarity		-0.95	-1.17	-0.66	-0.62	
DN987241	No significant similarity		-1.31	-1.18	-0.17	-1.36	
DN987153	No significant similarity	6.00E+00	-0.74	-1.18	1.21	-0.80	
DN988572	aspartic proteinase, Triticum aestivum	2.00E-53	-1.09	-1.19	-0.10	-0.94	
DN987005	No significant similarity	4.10E+00	-1.52	-1.19	-0.25	-0.80	
BQ826355	eukaryotic translation initiation factor 5, Zea mays	6.00E-58	-0.86	-1.19	-0.77	-0.79	
DN987093	No significant similarity		-1.64	-1.19	-0.07	-0.58	
DN986570	No significant similarity		-0.87	-1.20	-0.57	-0.32	
DN988842	No significant similarity		-0.83	-1.20	0.51	-0.10	
DN987969	ribosomal protein S8, Oryza sativa Japonica	9.00E-88	0.02	-1.20	1.60	0.50	
DN987228	No significant similarity		-0.70	-1.21	0.94	-0.41	
BG322346	MA3 domain-containing protein, Arabidopsis lyrata	1.00E-37	-2.02	-1.21	-0.66	-0.10	
DN987446	No significant similarity	3.10E-02	-1.28	-1.21	-1.40	-0.68	
DN987883	No significant similarity		-2.48	-1.22	-0.68	-0.72	
DN986914	No significant similarity	5.20E-01	-1.88	-1.22	-0.74	-0.96	
DN988116	No significant similarity		-2.22	-1.22	-0.62	-0.14	
DN985684	adenylate translocator, Arabidopsis thaliana	3.00E-07	-2.03	-1.23	-0.95	-1.00	
DN988884	No significant similarity		-1.34	-1.23	-0.02	0.21	
BQ826360	No significant similarity		-0.99	-1.23	-0.27	-0.95	
DN986662	No significant similarity	7.40E+00	-0.15	-1.23	-0.01	-0.63	
DN988395	No significant similarity		-1.74	-1.23	0.80	-0.70	
DN988882	phospholipid-translocating ATPase, Arabidopsis thaliana	2.00E-66	-0.91	-1.24	-1.21	-0.16	
DN987200	No significant similarity	4.30E+00	-0.65	-1.24	-0.49	-1.07	
DN988847	No significant similarity		-1.75	-1.24	-0.10	-0.07	
DN987314	putative cysteine proteinase inhibitor, Oryza sativa Japonica	2.00E-38	-1.00	-1.25	-0.63	-1.21	
DN987585	No significant similarity	5.10E+00	-0.72	-1.26	0.84	0.21	
BQ826400	NEDD8-conjugating enzyme Ubc12-like, Zea mays	5.00E-31	-0.62	-1.26	0.29	-0.36	
DN988879	No significant similarity		-0.80	-1.26	-0.56	0.00	

Acc #	Blast x Hits	E value	M2D	M28D	Z2D	Z28D	Expression Level
DN986672	No significant similarity		-1.40	-1.28	-1.49	-1.21	
DN987230	No significant similarity		-0.81	-1.29	0.30	-0.36	
BQ826378	No significant similarity		-0.61	-1.30	-0.26	-0.88	
DN987268	No significant similarity		-1.17	-1.30	-0.02	-1.09	
BG322304	HMG1/2-like protein, Hordeum vulgare	4.20E+00	-1.97	-1.30	-0.73	-0.44	
DN987002	No significant similarity		-0.96	-1.31	0.19	-0.57	
DN985503	No significant similarity		-1.60	-1.32	-1.13	0.14	
BG322349	No significant similarity	7.20E+00	-0.56	-1.33	-0.54	-1.18	
DN987258	No significant similarity	1.50E-01	-0.62	-1.34	0.05	-0.66	
DN987051	No significant similarity	2.40E-01	-0.99	-1.34	-0.54	-1.40	
DN987072	No significant similarity	5.00E+00	-0.39	-1.35	0.13	-1.51	
DN987262	No significant similarity		-0.32	-1.35	-0.03	-0.36	
DN987063	No significant similarity		-1.12	-1.35	-0.19	-0.70	
DN988925	ubiquitin-protein ligase, expressed, Triticum aestivum	1.00E-16	-1.73	-1.35	-0.60	-0.53	
DN985534	carbohydrate transporter/ sugar porter/ transporter, Zea mays	3.00E-29	-1.03	-1.36	-1.03	-1.35	
DN988405	No significant similarity		-0.58	-1.36	-0.75	-0.18	
BG322298	ATP binding protein, Ricinus communis	4.00E-36	-0.74	-1.37	-0.44	-0.82	
DN987034	No significant similarity	4.70E+00	-1.99	-1.37	-0.97	-1.58	
BQ826411	No significant similarity		-0.80	-1.37	0.09	-0.16	
DN985636	hypothetical protein LOC100276833, Zea mays	4.00E-04	-2.91	-1.39	-1.41	-0.65	
BQ826380	isovaleryl-CoA dehydrogenase, Beta vulgaris	4.60E-02	-1.20	-1.40	-0.78	-1.15	
BQ826365	aspartic proteinase, Oryza sativa	8.00E-31	-0.69	-1.40	-0.50	-0.88	
DN987253	No significant similarity	6.90E+00	-0.45	-1.41	1.14	-0.09	
BQ826374	hypothetical protein LOC100277472 [Zea mays] >gb|ACG40883.1| hypothetical protein [Zea mays]	1.00E-17	-0.63	-1.41	0.36	-0.27	
DN987166	No significant similarity	2.30E-01	-0.72	-1.42	1.21	0.52	
DN988755	No significant similarity		-1.21	-1.43	1.20	-0.04	
DN987165	No significant similarity	2.30E+00	-0.90	-1.44	-0.08	-0.69	
DN987094	No significant similarity	1.00E+00	-0.52	-1.45	0.37	-1.25	
DN987962	No significant similarity	2.80E-01	-0.78	-1.45	0.74	0.10	
DN986638	No significant similarity	9.80E-01	-1.47	-1.45	-0.94	-0.83	
DN987229	No significant similarity		-1.29	-1.46	0.01	-0.60	
DN987049	No significant similarity	5.60E-01	-1.25	-1.46	-0.35	-0.60	
DN987040	protein kinase C inhibitor-like protein, Arabidopsis thaliana	2.00E-55	0.10	-1.47	0.32	-1.39	
BQ826309	RNA polymerase-associated protein RTF1, Ricinus communis	2.00E-31	-1.93	-1.48	-0.60	-0.38	
DN987771	ribosomal protein S8, Oryza sativa Japonica	3.00E-68	-0.03	-1.49	1.30	0.22	
DN985584	glyceraldehyde-3-phosphate dehydrogenase, cytosolic 1, Zea mays	5.00E-95	-0.62	-1.52	0.38	-0.22	
DN988837	No significant similarity		-1.15	-1.54	1.21	0.07	
DN986527	No significant similarity		-0.54	-1.54	-0.36	-0.67	
DN985562	No significant similarity	8.80E-01	-2.07	-1.55	-0.76	-1.45	
BQ825981	No significant similarity	2.20E+00	-1.42	-1.55	-0.74	-0.96	
DN986717	No significant similarity	2.30E+00	-0.78	-1.55	0.45	-0.65	
BQ826304	60S ribosomal protein L5-1Zea mays	1.00E-03	-1.91	-1.56	-0.91	-0.65	
DN986963	No significant similarity		-1.28	-1.56	-1.28	-0.90	
DN986945	No significant similarity		-0.46	-1.57	-0.37	-1.38	
DN986654	No significant similarity		-1.31	-1.58	-0.66	-0.36	
DN985406	No significant similarity	2.60E-01	-0.94	-1.58	-0.60	-0.72	
DN987293	No significant similarity	8.70E-01	-1.96	-1.59	-1.52	-1.37	
DN989106	retrotransposon protein, Oryza sativa Indica	5.00E-04	-1.83	-1.61	-0.62	-0.71	
DN985680	No significant similarity		-2.90	-1.63	-2.63	-1.49	
DN987109	No significant similarity	2.40E+00	-0.11	-1.64	-0.08	-0.42	
DN987440	ORF64c, Arabidopsis lyrata	1.00E-10	-2.13	-1.64	-1.08	-0.61	
DN986640	No significant similarity		-1.54	-1.64	-1.98	-1.28	
DN987175	No significant similarity	3.30E+00	-0.29	-1.64	0.06	-0.22	
DN987856	No significant similarity	5.20E-01	-1.24	-1.65	0.53	-0.68	
DN987449	No significant similarity	6.00E-02	-0.28	-1.65	-0.31	-0.68	
DN987011	No significant similarity	6.10E+00	-0.42	-1.65	0.26	-0.15	
DN987302	legumain-like protease, Zea mays	4.00E-29	-3.15	-1.65	-0.38	-0.40	
DN987242	No significant similarity		-0.15	-1.66	1.20	-0.09	
DN987256	No significant similarity		-0.88	-1.69	-0.10	-0.56	
BQ826402	No significant similarity		-2.14	-1.70	-0.27	-0.62	

Acc #	Blast x Hits	E value	M2D	M28D	Z2D	Z28D
DN987106	No significant similarity	4.10E+00	-1.06	-1.71	0.72	-1.22
DN986014	No significant similarity	2.10E+00	-2.88	-1.71	-0.99	-0.84
DN987010	No significant similarity		-0.81	-1.71	-0.07	-0.10
DN987272	No significant similarity		-0.80	-1.72	-0.79	-1.36
BQ825945	No significant similarity		-1.66	-1.73	0.09	-1.20
DN988471	No significant similarity	8.90E+00	-0.73	-1.73	0.14	-1.46
DN985498	No significant similarity	8.90E+00	-1.86	-1.73	-1.38	-1.89
BQ826431	protein translation factor SUI1, Zea mays	1.00E-37	-0.92	-1.73	-1.60	-0.01
DN985663	No significant similarity		-2.39	-1.74	-1.48	-1.06
DN985439	No significant similarity		-2.08	-1.74	-1.74	-1.43
DN985426	SAP domain containing protein, expressed, Oryza sativa Japonica	1.00E-28	-1.41	-1.75	-1.47	-1.44
DN986953	No significant similarity	8.00E+00	-1.86	-1.76	-1.90	-1.61
DN987150	No significant similarity		-1.34	-1.77	-0.48	-1.03
DN986956	No significant similarity	4.90E-01	-1.16	-1.77	-0.01	-0.96
DN987154	attachment glycoprotein, Avian metapneumovirus	4.00E-03	-1.13	-1.77	0.26	-0.52
DN989105	hypothetical protein SORBIDRAFT_0057s002150, Sorghum bicolor	2.00E-60	-1.22	-1.77	0.68	-0.46
DN986968	No significant similarity	4.10E-01	-2.55	-1.78	-0.91	-0.03
DN987259	No significant similarity		-0.98	-1.78	-0.49	-0.73
DN987074	No significant similarity	8.70E+00	-2.62	-1.80	-0.48	-0.18
DN985545	Os12g0516200, Oryza sativa Japonica	4.00E-12	-1.56	-1.80	-0.78	-1.02
DN987055	No significant similarity	1.80E-01	-1.00	-1.80	-1.63	-2.31
DN987690	ferrochelatase-2,Zea mays	1.00E-10	-1.23	-1.81	0.60	-0.58
BQ825994	No significant similarity	2.10E+00	-1.09	-1.82	0.14	0.08
DN986975	No significant similarity		-1.46	-1.83	1.15	-1.65
DN986532	No significant similarity		-1.20	-1.85	-0.37	-2.12
DN986588	No significant similarity		-1.05	-1.85	1.00	-0.80
DN987244	No significant similarity	2.20E+00	-0.77	-1.87	-0.09	-0.61
DN987157	No significant similarity		-0.34	-1.88	-0.12	-1.30
DN987177	No significant similarity		-1.91	-1.90	-0.97	-1.86
DN987247	Pseudouridine synthase, Rsu, Burkholderia glumae	8.00E-09	-1.48	-1.90	-0.18	-0.50
DN986794	No significant similarity	3.80E-01	-3.35	-1.92	-2.25	-1.96
DN987208	No significant similarity	1.40E+00	-1.49	-2.00	-0.13	-1.23
DN987015	No significant similarity		-1.15	-2.05	-0.36	-1.44
BQ826371	secreted hydrolase [Saccharopolyspora erythraea NRRL 2338] >emb|CAM04444.1| secreted hydrolase [Saccharopolyspora erythraea NRRL 2338]	6.60E-01	-0.75	-2.05	-0.05	-0.63
DN987223	No significant similarity		-1.74	-2.06	0.27	-1.19
DN985788	No significant similarity	8.50E+00	-0.98	-2.08	-0.02	-0.55
DN987171	No significant similarity	4.10E+00	-1.58	-2.13	0.00	-0.89
DN987144	No significant similarity		-0.56	-2.15	-0.13	-1.15
DN985661	No significant similarity	9.90E+00	-0.94	-2.15	0.00	-1.61
DN986659	No significant similarity	3.70E+00	-0.87	-2.15	-1.28	-2.00
DN987297	catalase catA-like protein, Oryza sativa Japonica	8.00E-24	-1.86	-2.16	0.98	-0.85
DN987118	RNA polymerase beta'' subunit, partial, Zea mays	7.00E-72	-0.21	-2.18	1.55	-1.06
DN986674	No significant similarity	3.40E-01	-0.80	-2.19	-1.53	-0.47
DN987191	No significant similarity	9.20E-02	-0.98	-2.21	-0.64	-2.07
DN987120	No significant similarity	7.60E-01	-0.16	-2.22	-0.13	-0.99
DN987266	No significant similarity	6.10E+00	-1.10	-2.25	-0.33	0.14
DN987148	No significant similarity	2.30E+00	-0.38	-2.27	1.55	-0.09
DN987249	No significant similarity	5.90E-02	-1.22	-2.30	0.39	-0.56
DN987151	No significant similarity		-1.59	-2.36	1.16	-0.39
BQ826426	universal stress protein 23267, Hordeum vulgare	4.00E-18	-1.68	-2.37	-0.26	-1.66
DN987222	No significant similarity		-2.39	-2.42	-0.54	-1.23
DN987019	No significant similarity		-2.16	-2.45	-1.27	-1.31
DN985683	No significant similarity	4.00E+00	-1.17	-2.47	0.28	-1.23
DN987045	No significant similarity	2.40E-01	-1.72	-2.49	-1.06	-1.42
DN988688	No significant similarity		-2.13	-2.49	-1.49	-1.75
BQ826377	No significant similarity		-1.26	-2.51	0.88	-0.39
DN986667	No significant similarity	1.90E+00	-2.65	-2.53	-1.84	-2.41

Expression Level

Expression Level

Acc #	Blast x Hits	E value	M2D	M28D	Z2D	Z28D
DN985957	No significant similarity		-1.58	-2.55	-1.12	-1.74
DN987001	No significant similarity		-2.40	-2.56	-0.57	-0.67
DN986783	No significant similarity	4.70E+00	-1.59	-2.56	0.63	-0.70
DN988526	No significant similarity		-1.93	-2.57	0.91	-1.00
DN986673	No significant similarity	1.40E+00	-1.87	-2.60	-1.55	-2.02
DN988442	No significant similarity		-2.65	-2.62	-1.48	-1.23
DN987304	No significant similarity	1.50E+00	-0.14	-2.63	0.88	-1.44
DN988013	No significant similarity	7.50E+00	-2.68	-2.64	-2.09	-1.45
DN987273	No significant similarity		-1.59	-2.64	0.42	-1.33
DN987047	hypothetical protein IscW_ISCW007425, Ixodes scapularis	4.00E-04	-2.28	-2.65	-0.10	-0.48
DN986671	No significant similarity		-0.46	-2.65	0.69	-0.72
DN988165	No significant similarity	7.70E-01	-2.71	-2.65	1.10	-0.81
DN985850	No significant similarity	1.70E+00	-2.28	-2.65	-1.27	-1.87
DN987184	No significant similarity	3.80E+00	-1.37	-2.66	0.89	-0.91
DN986685	No significant similarity	2.40E+00	-1.71	-2.69	-0.28	-1.92
DN985649	No significant similarity		-1.97	-2.71	1.34	-0.85
DN985666	No significant similarity	7.70E+00	-1.44	-2.73	0.54	-0.92
DN987028	glyoxylase1, Zea mays	9.00E-28	-1.92	-2.76	-0.77	-1.86
DN986655	No significant similarity	3.40E+00	-2.69	-2.79	-1.94	-2.44
DN986960	No significant similarity	3.60E-01	-1.70	-2.79	-1.14	-1.64
DN987289	No significant similarity	3.40E+00	-1.81	-2.81	-1.22	-2.09
DN985519	No significant similarity	1.10E+00	-2.70	-2.81	-1.14	-1.67
DN987145	No significant similarity		-0.20	-2.82	-0.31	-1.30
DN985568	No significant similarity		-1.07	-2.86	0.43	-1.05
DN985766	No significant similarity		-2.27	-2.97	-2.03	-2.66
DN986776	No significant similarity		-2.07	-2.98	-1.24	-1.91
DN986538	No significant similarity	6.00E-01	-0.88	-2.99	-0.45	-0.72
DN986670	No significant similarity	6.20E+00	-3.21	-3.00	-1.46	-1.49
DN987038	No significant similarity	3.30E-01	-1.43	-3.05	0.47	-1.33
DN987213	No significant similarity		-1.30	-3.06	0.23	-1.03
DN985574	No significant similarity	9.60E-01	-3.11	-3.06	-1.11	-1.42
DN987255	No significant similarity	4.70E+00	-2.25	-3.10	-1.76	-2.53
DN985656	No significant similarity	1.30E+00	-3.06	-3.13	-1.45	-1.12
DN987003	No significant similarity	9.40E+00	-2.91	-3.14	-0.24	-0.06
DN985512	No significant similarity		-0.71	-3.15	-0.43	0.04
DN987212	No significant similarity		-2.24	-3.18	-0.76	-1.67
DN987042	No significant similarity	1.10E+00	-2.30	-3.25	0.79	-1.00
DN987142	No significant similarity	1.60E+00	-1.97	-3.30	-1.53	-2.02
DN986643	No significant similarity		-3.49	-3.32	-2.33	-2.02
DN986651	No significant similarity	1.60E-01	-1.59	-3.35	0.77	-0.48
DN987032	No significant similarity	1.70E+00	-1.93	-3.38	-0.69	-2.06
DN985517	No significant similarity	4.90E+00	-3.41	-3.39	-1.81	-2.01
DN985696	No significant similarity		-1.82	-3.41	0.74	-0.75
DN987299	No significant similarity		-0.42	-3.43	-2.75	-1.32
DN987480	dehydrin DHN1, Zea mays	7.00E-11	-2.32	-3.45	-1.74	-2.43
DN987172	No significant similarity	6.30E-02	-2.08	-3.46	0.84	-0.60
DN985622	No significant similarity		-3.35	-3.47	-2.18	-2.25
DN985664	No significant similarity	7.70E+00	-0.61	-3.56	-0.21	-1.36
DN987315	No significant similarity	8.30E+00	-3.03	-3.58	-1.68	-1.90
DN986951	No significant similarity	3.80E+00	-2.54	-3.83	-1.83	-2.46
DN987224	No significant similarity	1.10E+00	-1.29	-3.98	-1.46	-2.34
DN985448	No significant similarity	9.80E-01	-2.13	-4.01	-0.93	-2.67
DN988851	No significant similarity	3.00E+00	-2.66	-4.03	-1.86	-2.58
DN985598	No significant similarity	1.50E+00	-4.02	-4.05	-1.22	-1.30
DN987206	No significant similarity		-3.84	-4.16	-0.92	-1.36
DN988458	No significant similarity		-1.15	-4.29	-0.02	-1.40
DN987116	No significant similarity		-2.25	-4.32	-0.74	-2.67
DN987250	No significant similarity	1.60E+00	-2.03	-4.36	-0.55	-2.63
DN987017	No significant similarity		-3.38	-4.83	-2.09	-2.73
DN986872	No significant similarity	8.60E-01	-3.78	-4.84	-1.93	-2.33
DN987203	No significant similarity		-3.31	-5.13	-1.94	-2.71
DN987202	No significant similarity		-4.88	-5.14	-2.72	-2.44
DN987053	No significant similarity	1.10E-01	-4.56	-5.27	-2.27	-3.22
DN987043	No significant similarity	1.30E+00	-3.39	-5.34	-0.59	-2.92
