# Supplementary material for: Transcriptional Analysis of Resistance to Low Temperatures in Bermudagrass Crown Tissues
Source: PLoS One. 2015 Sep 8;10(9):e0136433. doi: 10.1371/journal.pone.0136433 (PMC4562713; doi:10.1371/journal.pone.0136433)
Supplement: S2 Table — (PDF) [file pone.0136433.s003.pdf]

Acc #	NCBI Matches	Cluster	M2D	M28D	Z2D	Z28D	Expression Level	
BQ826386	Senescence-associated protein DIN1, Zea Mays	1	6.94	5.99	4.83	3.87	5 to 7	14.63323
BG322297	AAA-type ATPase family protein, Oryza sativa Japonica	2	3.28	3.17	1.79	1.50	3 to 5	
BQ826306	Sucrose synthase metabolism, Oryza sativa Indica	2	3.11	2.93	2.14	1.34	1 to 3	
BQ826366	Actin, Picea mariana	2	2.21	2.28	1.37	1.64	-1 to 1	
BQ826101	ADP-ribosylation factor, Pinus pinaster	2	2.15	2.22	1.65	1.37	-1 to -3	
BQ826356	Protein kinase (AME2/AFC1), Oryza sativa Japonica	2	1.35	2.14	1.12	2.03	-3 to -5	
DN989174	Ribosomal protein S1, Sorghum bicolor	2	1.76	2.10	0.92	2.64		
BQ826239	Beta-1,3-glucanase,Oryza sativa	3	1.29	1.69	0.34	-0.08		
BQ825943	Ribosomal protein L11, Felis catus	3	1.59	1.59	0.52	-0.45		
DN988980	Ferrochelatase-2, Zea mays	3	1.07	1.10	0.80	0.51		
BQ826337	Ribosomal protein S15, Triticum aestivum	3	1.52	1.10	1.45	0.75		
DN987924	bHLH transcription factor-like protein, Oryza sativa Japonica	3	0.90	1.04	0.28	-0.08		
BQ825934	Acyl-CoA-binding protein, Panax ginseng	3	3.00	1.00	1.87	-0.32		
BG322336	GRAS family transcription factor, Populus trichocarpa	3	1.97	0.86	-0.12	0.18		
BQ826279	Sucrose synthase, Sorghum bicolor	3	1.40	0.41	-0.14	0.17		
BQ826352	Anthranilate phosphoribosyltransferase-like protein, Arabidopsis thaliana	3	1.61	0.30	0.78	0.53		
DN989089	BRASSINOSTEROID INSENSITIVE 1-associated receptor kinase 1, Zea mays	4	0.82	2.78	0.48	0.57		
DN987085	Aspartate aminotransferase, Triticum aestivum	4	0.71	2.26	0.61	0.91		
DN987459	Dehydrin 13, Zea mays	4	1.12	1.92	1.06	1.31		
BQ826329	ATP synthase subunit 6, Phaeosphaeria nodorum SN15	4	0.39	1.56	-0.53	0.51		
DN986703	Brahma-associated protein 111kD, Drosophila melanogaster	4	0.10	1.54	-0.76	1.01		
DN987620	Senescence-associated protein, Lilium longiflorum	4	0.70	1.47	0.61	1.56		
DN987650	Ribosomal protein S8, Oryza sativa Japonica	4	-0.39	1.40	0.10	1.87		
DN988370	Senescence-associated protein, Pisum sativum	4	0.91	1.25	0.25	0.69		
DN988922	RNA polymerase beta' subunit, Sorghum bicolor	4	1.11	1.24	0.86	1.18		
BQ826357	Phenylalanine ammonia-lyase, Zea mays	4	0.82	1.20	0.82	1.00		
DN989054	Senescence-associated protein, Picea abies	4	0.47	1.20	1.10	0.78		
BQ826072	Hypothetical protein PTT_16148, Pyrenophora teres	4	1.33	1.15	1.04	1.21		
DN987779	Senescence-associated protein, Lilium longiflorum	4	0.46	1.09	0.38	1.06		
DN987809	Senescence-associated protein, Lilium longiflorum	4	0.70	1.09	0.10	0.46		
DN987196	Elastin binding protein EbpS, Staphylococcus lugdunensis HKU09-01	4	0.47	1.08	0.66	1.36		
DN986445	Polyprotein, Bermuda grass mosaic virus	4	0.51	1.08	0.60	1.34		
BQ825936	Nucleolar GTP-binding protein, Oryza sativa Japonica	4	0.75	0.98	0.62	1.19		
BQ825958	Microtubule-associated protein MAP65-1a, Oryza sativa	4	0.88	0.93	0.92	1.29		
DN988333	Senescence-associated protein, Pyrus communis	4	0.09	0.79	1.12	1.40		
BQ826445	LIM domain protein GLIM1a, Populus tremula	4	0.62	0.71	0.73	1.01		
DN985606	Xylanase inhibitor protein 1, Zea mays	4	0.15	0.66	-0.10	1.02		
BQ826412	DnaJ protein, Oryza sativa Japonica	4	-0.01	0.55	0.69	2.22		
BG322311	Ascorbate peroxidase, Hordeum vulgare	5	-0.22	0.24	0.99	1.58		
DN988483	CTP synthase , Zea mays	5	-0.36	-0.51	0.68	1.55		
DN987932	CBL-interacting serine/threonine-protein kinase 1, Zea mays	5	0.26	0.08	1.04	1.47		
DN987474	Adenosylhomocysteinase,putative,expressed, Oryza sativa Japonica	5	-0.40	-0.19	1.04	1.36		
DN987274	Cation transport protein chaC, Zea mays	5	0.15	0.20	0.12	1.21		
BQ826267	60S ribosomal protein L37, Zea mays	5	0.74	0.26	0.92	1.11		
BQ826365	Aspartic proteinase, Oryza sativa	5	-1.49	-0.35	0.63	1.09		
DN986971	Cysteine proteinase 1 precursor, Zea mays	5	-0.79	0.44	-0.11	1.04		
DN987448	Auxin-induced in root cultures protein 12 precursor, Ricinus communis	5	0.33	-0.39	1.68	0.64		
DN987352	Inverted formin-2, Pan troglodytes	5	-0.16	-0.21	1.44	0.60		
DN987058	CBL-interacting protein kinase 25, Sorghum bicolor	5	0.02	-1.20	1.60	0.50		
DN987408	Hypothetical protein CJBH_1340, Campylobacter jejuni	5	-0.03	-1.49	1.30	0.22		
BG322307	RabGAP/TBC domain-containing protein, Arabidopsis thaliana	5	0.19	0.10	1.14	0.00		
DN987102	Ketol-acid reductoisomerase, Zea mays	5	0.93	-1.03	1.68	-0.10		
BQ826385	40S ribosomal protein S11, Zea mays	6	0.93	0.75	-0.17	-1.46		
BG322354	Elongation factor 1-gamma 3, Oryza sativa Indica	6	0.84	0.27	0.45	-1.25		
DN988344	Senescence-associated protein, Lilium longiflorum	6	0.95	1.30	-0.65	-1.03		
BG322345	Ubiquitin-conjugating enzyme family protein,expressed, Oryza sativa Japonica	7	-0.21	-2.18	1.55	-1.06		
DN985426	SAP domain containing protein,expressed, Oryza sativa Japonica	7	-1.86	-2.16	0.98	-0.85		
BG322298	ATP binding protein,putative, Ricinus communis	7	-1.48	-1.90	-0.18	-0.50		
DN988882	Phospholipid-translocating ATPase, Arabidopsis thaliana	7	-1.23	-1.81	0.60	-0.58		
DN985397	Ribosomal protein S3, Leersia tisserantii	7	-0.62	-1.52	0.38	-0.22		
BQ826344	Ubiquitin-like protein, Triticum aestivum	7	0.10	-1.47	0.32	-1.39		
BG322357	F-box domain containing protein, Zea mays	7	-0.69	-1.40	-0.50	-0.88		
DN988920	Autophagy-related protein 8 precursor, Zea mays	7	-0.74	-1.37	-0.44	-0.82		
DN987399	Elongation factor 1-alpha, Zea mays	7	-0.62	-1.26	0.29	-0.36		
DN988572	Aspartic proteinase, Triticum aestivum	7	-1.00	-1.25	-0.63	-1.21		
DN988512	CorA-like Mg2+ transporter protein, Oryza sativa Japonica	7	-0.86	-1.19	-0.77	-0.79		
BG322346	MA3 domain-containing protein, Arabidopsis lyrata	7	-1.09	-1.19	-0.10	-0.94		
DN985458	Cysteine proteinase, Hordeum vulgare	7	-0.42	-1.04	-0.33	-0.52		
DN987065	MtN19-like protein, Arabidopsis thaliana	7	-0.95	-1.02	0.03	-1.19		
DN985684	Adenylate translocator, Arabidopsis thaliana	7	-1.14	-0.75	-0.35	-0.76		
DN987969	Ribosomal protein S8, Oryza sativa Japonica	7	-1.07	-0.66	-0.25	-0.95		
DN987028	Glyoxylase1, Zea mays	8	-3.05	-0.12	-2.05	0.15		
DN987297	Catalase catA-like protein, Oryza sativa Japonica	8	-2.02	-1.21	-0.66	-0.10		
DN987247	Pseudouridine synthase,Rsu, Burkholderia glumae	8	-1.93	-1.48	-0.60	-0.38		
DN989106	Retrotransposon protein, Oryza sativa Indica	8	-1.73	-1.35	-0.60	-0.53		
DN985584	Glyceraldehyde-3-phosphate dehydrogenase,cytosolic 1, Zea mays	8	-1.70	-0.97	-1.25	-0.14		
BQ826309	RNA polymerase-associated protein RTF1,putative, Ricinus communis	8	-1.63	-0.90	-0.40	-0.27		

DN987040	Protein kinase C inhibitor-like protein, Arabidopsis thaliana	8	**-1.53**	**-0.07**	**0.08**	**0.57**
DN988925	Ubiquitin-protein ligase, Triticum aestivum	8	**-1.39**	**-0.13**	**-0.36**	**-0.05**
BQ826400	NEDD8-conjugating enzyme Ubc12-like, Zea mays	8	**-1.32**	**-0.34**	**-1.24**	**-0.75**
DN987314	Cysteine proteinase inhibitor, Oryza sativa Japonica	8	**-1.29**	**-0.23**	**-0.38**	**-0.28**
DN985524	Delta 1-pyrroline-5-carboxylate synthetase 1, Sorghum bicolor	8	**-0.99**	**-0.34**	**-1.05**	**-0.72**
DN988922	RNA polymerase beta' subunit, Sorghum bicolor	8	**-0.92**	**-1.73**	**-1.60**	**-0.01**
DN986977	Arginyl-tRNA synthetase, Zea mays	8	**-0.91**	**-1.24**	**-1.21**	**-0.16**
DN987897	Aspartic proteinase oryzasin-1, Zea mays	8	**-0.69**	**-0.88**	**-1.26**	**-0.38**
DN987494	NADP-dependent malic enzyme, Zea mays	8	**-0.49**	**0.19**	**-1.03**	**0.24**
DN988453	Phosphoinositide phosphatase, Oryza sativa Japonica	9	**-0.76**	**0.26**	**-2.91**	**-1.25**
DN988543	von Willebrand factor type A domain containing protein, Oryza sativa Japonica	9	**0.68**	**0.68**	**-1.74**	**-2.34**
BQ826426	Universal stress protein 23267, Hordeum vulgare	10	**-2.32**	**-3.45**	**-1.74**	**-2.43**
DN987690	Ferrochelatase-2, Zea mays	10	**-1.92**	**-2.76**	**-0.77**	**-1.86**
DN987771	Ribosomal protein S8, Oryza sativa Japonica	10	**-1.68**	**-2.37**	**-0.26**	**-1.66**
DN985534	Carbohydrate transporter/ sugar porter/ transporter, Zea mays	10	**-1.41**	**-1.75**	**-1.47**	**-1.44**
DN987480	Dehydrin DHN1, Zea mays	10	**-3.15**	**-1.65**	**-0.38**	**-0.40**
DN987302	Legumain-like protease, Zea mays	10	**-1.83**	**-1.61**	**-0.62**	**-0.71**
