# Supplementary material for: Transcriptional Analysis of Resistance to Low Temperatures in Bermudagrass Crown Tissues
Source: PLoS One. 2015 Sep 8;10(9):e0136433. doi: 10.1371/journal.pone.0136433 (PMC4562713; doi:10.1371/journal.pone.0136433)
Supplement: S3 Table — (PDF) [file pone.0136433.s004.pdf]

RT-PCR analyses of selected cold resistant genes and their comparison with microarray expression.

	NCBI	MSU2		Zebra 2		MSU 28		Zebra 28	
Gene	Accession #	Microarray[a]	qRT-PCR[b]	Microarray[a]	qRT-PCR[b]	Microarray[a]	qRT-PCR[b]	Microarray[a]	qRT-PCR[b]
Sucrose Synthase 2 (*Zea mays*)	BQ826306	3.11	3.51	1.33	2.09	2.48	2.92	2.67	2.53
Sucrose Synthase 2 (*Sorghum bicolor*)	BQ826279	1.59	1.55	0.51	1.03	1.58	1.73	-0.44	0.59
Acyl CoA Binding Protein	BQ825934	0.82	0.63	0.48	0.94	2.78	3.56	0.57	0.83
Protein Kinase (AME2/AFC1)	BQ826356	0.39	0.84	-0.53	0.31	1.56	1.23	0.50	0.77

[a] values represent the $\log_2$ ratio of expression under cold acclimation vs control conditions.

[b]values represent $\log_2$ ratio of $\Delta\Delta C^T$ values from qRT-PCR data.
